# Quantification of aflatoxins and sterigmatocystin in walnuts, cashews, pistachios, peanuts, and hazelnuts by using UHPLC-MS/MS following *Aspergillus flavus* inoculation

**DOI:** 10.1007/s12550-026-00666-w

**Published:** 2026-08-04

**Authors:** Sarah Schneidemann-Bostelmann, Franka Nöth, Stefan Asam, Michael Rychlik

**Affiliations:** Chair of Analytical Food Chemistry, Technical University of Munich, Freising, Germany

**Keywords:** multi mycotoxins, aflatoxins, sterigmatocystin, *Apergillus flavus*, inoculation, tree nuts, SIDA, LC-MS/MS.

## Abstract

Nuts are widely consumed worldwide and valued for their high nutritional quality. Tree nuts are, however, prone to colonization by various fungal genera that can cause spoilage and lead to the formation of toxic secondary metabolites, among which mycotoxigenic *Aspergillus* (A.) species are of particular concern because they produce hepatotoxic aflatoxins (AFs). In this study, untreated, shelled walnuts, hazelnuts, cashews, pistachios, and peanuts were surface-sterilized, inoculated with different *A. flavus* strains and incubated under controlled conditions. The concentrations of AFs and sterigmatocystin (STC) in the edible kernels were quantified by an ultra-high-performance liquid chromatography method, coupled with tandem mass spectrometry (UHPLC-MS/MS). In walnuts inoculated with five *A. flavus* strains, toxin production was highly strain dependent: four strains produced AFB1 in a wide range from 0.18 µg/kg to > 11,000 µg/kg, three strains formed AFB2 (3.58-1,411 µg/kg), and three strains synthesized STC (0.26–262 µg/kg), whereas one strain did not generate any AFs or STC, and none of the strains produced AFG1 or AFG2. In pistachios, inoculation with strains AF70 and CBS119.62 did not result in detectable AF formation, in contrast to hazelnuts, cashews, and peanuts, on which these strains yielded measurable toxin levels (AFB1: hazelnuts 0.11–3.56 µg/kg, cashews 0.15–0.16 µg/kg, peanuts 0.06–17.9 µg/kg; AFB2: hazelnuts 0.13–0.18 µg/kg, cashews < LOQ, peanuts 0.26–7.59 µg/kg; STC: hazelnuts 0.08 µg/kg, peanuts 5.28 µg/kg). Overall, markedly higher concentrations of *A. flavus* toxins were detected in walnuts than in the other nut types, identifying walnuts as a particularly susceptible matrix and indicating that AF and STC contamination of walnuts may pose an increasing food-safety challenge under future climate-change scenarios.

**Supplementary Information:**

The online version contains supplementary material available at 10.1007/s12550-026-00666-w.

## Introduction

Nuts are widely consumed throughout the world and are valued for their high nutritional quality. They are rich sources of proteins, essential amino acids, unsaturated fatty acids, vitamins, and minerals, making them among the most nutrient-dense foods in the human diet (Škrbić et al., 2014; Cunha et al., 2018; Ebrahimi et al., 2022). Although from a botanical perspective, cashew kernels, pistachios, and peanuts are not classified as true nuts, the term “nut” is widely used in common language. For the sake of clarity and consistency, the collective term “nut” is therefore applied throughout this paper to all matrices investigated, namely cashews, hazelnuts, peanuts, walnuts, and pistachios.

The growing importance of this product category in the German market is reflected in several figures. The agricultural area dedicated to tree nut cultivation in Germany amounted to only 600 hectares in 2018 but had already increased to 1,600 hectares by 2024 (Statistisches Bundesamt, 2025). In the 2024/25 period, the consumption of nuts and dried fruits in Germany reached 482 thousand tons (Bundesanstalt für Landwirtschaft und Ernährung, 2025). Correspondingly, the German nut market generated sales of approximately €3.04 billion in 2024, a figure projected to rise to nearly €3.9 billion by 2030 (Statista, 2025a).

Tree nuts are susceptible to colonization by various fungal genera that may cause spoilage or lead to the formation of toxic secondary metabolites. Surveys of fungal incidence have consistently identified *Aspergillus* (A.) as the predominant genus associated with nut contamination, followed by *Rhizopus* and *Penicillium* (Bayman et al., 2002, Molyneux et al., 2007). Of particular concern are mycotoxigenic *Aspergillus* species, as they are responsible for the production of hepatotoxic aflatoxins (AF). Monitoring studies on walnuts further confirm that *Aspergillus* spp. represent the most frequently isolated fungi, with *A. flavus* being the dominant species detected (Bayman et al., 2002, Habibipour et al., 2016).

**Fig. 1:**
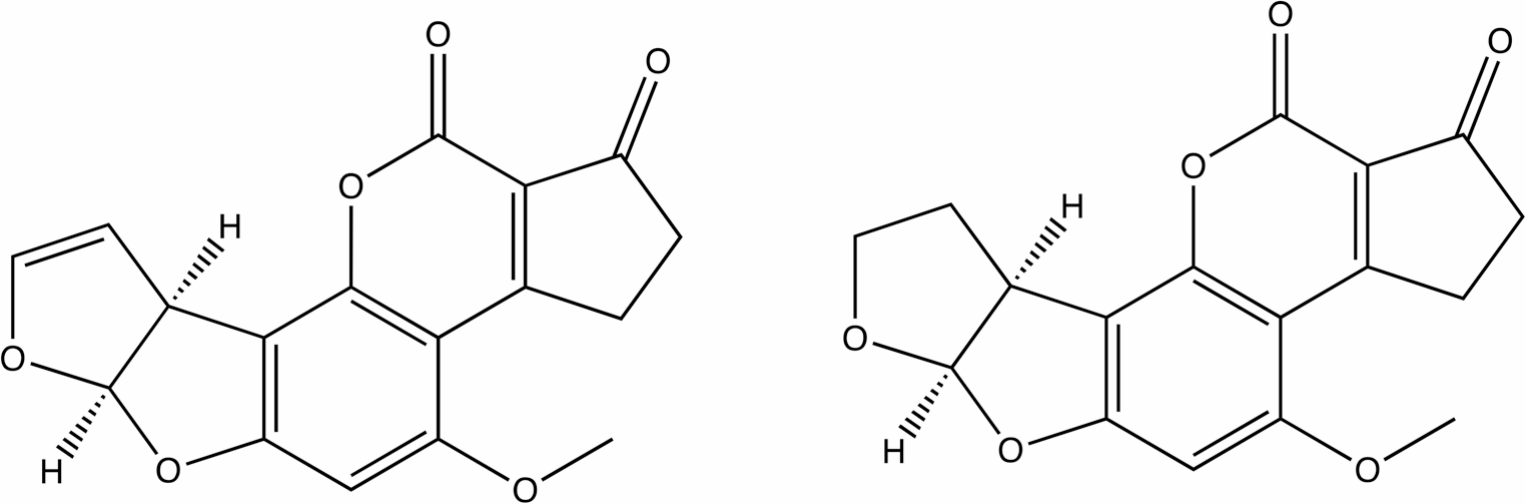
Structure of AFB1 (left) and AFB2 (right)

**Fig. 2:**
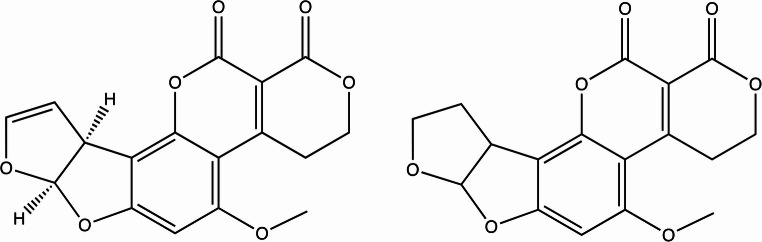
Structure of AFG1 (left) and AFG2 (right)

AFs are a class of extremely potent toxic compounds that are synthesized by certain molds within the *Aspergillus* genus, most notably *A. flavus* and *A. parasiticus* (Turner et al., 2009). These fungi are predominantly found in tropical and subtropical regions, where warm temperatures and high humidity favor its growth and AF production (Yohannis et al., 2025; Norlia et al., 2019). Four major AFs occur naturally - AFB1, AFB2, AFG1, and AFG2 - all of which are difuranocoumarin derivatives with closely related chemical structures. AFB1 and AFB2 contain a cyclopentane ring in their molecular framework, while AFG1 and AFG2 are characterized by the presence of a lactone ring (Figs. 1 and 2). AF have become a major focus of scientific investigation due to their strong carcinogenic properties and well-documented acute and long-term toxic effects on the human liver (Nakai et al., 2008; Abrar et al., 2013; EFSA et al., 2020). Among them, AFB1 has been classified by the International Agency for Research on Cancer (IARC) as carcinogenic to humans (Group 1) (IARC 2012). In response to the health risks posed by these compounds, the European Union (EU) has set maximum residue levels (MRLs) for AF in various nuts. For peanuts and tree nuts intended for direct human consumption, the MRLs are set at 2.0 µg/kg for AFB1 and 4.0 µg/kg for the sum of AFB1, AFB2, AFG1, and AFG2. Higher MRLs apply to pistachios, with 8.0 µg/kg for AFB1 and 10.0 µg/kg for total AFs, while for hazelnuts the MRLs are defined as 5.0 µg/kg for AFB1 and 10.0 µg/kg for the sum of AFs (European Commission, 2023).

**Fig. 3:**
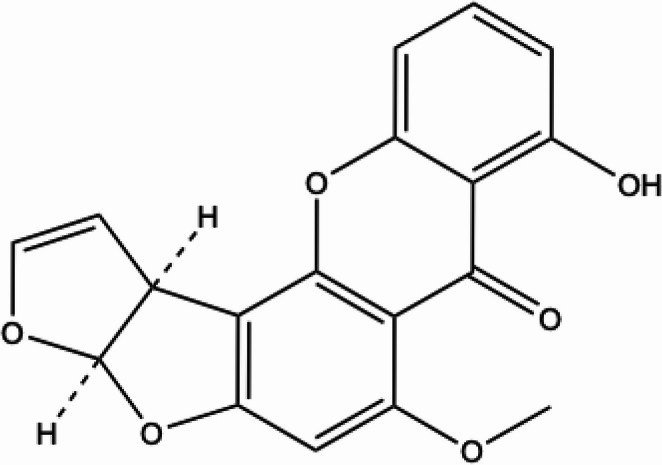
Structure of STC

A well-known biosynthetic precursor to AFB1 is sterigmatocystin (STC) (Fig. 3) (Yu et al., 2004). Its synthesis is limited to species within four sections of the genus *Aspergillus*: *Ochraceorosei*, *Versicolores*, *Nidulantes*, and *Flavi* (Rank et al., 2011). STC is classified as a possible human carcinogen (Group 2B) by the IARC (IARC, 1987) and has been shown to exhibit immunotoxic and immunomodulatory effects (Liu et al., 2014), as well as mutagenic activity (Gao et al., 2015). Unlike AFs, no legal maximum limits for STC are specified in regulation (EU) 2023/915 (European Commission, 2023).

The significance of mycotoxins, and AFs in particular, in nuts and nut products is further emphasized by data reported in the Annual Report of the Alert and Cooperation Network of the European Commission, which integrates data from the Rapid Alert System for Food and Feed (RASFF) and the Administrative Assistance and Cooperation (AAC) networks. In 2024, nuts and nut products accounted for 7% of all notifications, making them the second most reported product category. Of these notifications concerning this product group, 53% were attributable to mycotoxins, by far the most frequent reason for alerts on nuts and nut products (European Commission, 2025). This underscores a consistent trend from previous years. In 2023, mycotoxins in nuts, nut products, and seeds already ranked among the top notified hazards (European Commission, 2024). Pistachios are particularly susceptible to AF contamination. For example, in 2019, the German Federal Office of Consumer Protection and Food Safety (BVL) found that approximately 10% of the analysed pistachios (11/104) were contained with AFB1, with three samples exceeding the regulatory limit. In contrast, no AFs were quantifiable in any of the 77 analysed walnuts (BVL, 2019). A similar pattern was observed in 2013 (14/98 pistachios and 0/47 walnuts contaminated) (BVL, 2013). This trend is also supported by other sources: According to an European Food Safety Authority (EFSA) survey (EFSA 2007), pistachios showed the highest frequency and levels of AF contamination among hazelnuts, pistachios, peanuts, and cashews. As previously noted, several studies have examined the identification and characterization of microorganisms associated with nuts (Habibipour et al., 2016, Bayman et al., 2002). Beyond this, multiple investigations have documented and quantified the occurrence of AF and other mycotoxins across different nut types and nut-based products (Adaya-González et al., 2015; Cunha et al., 2018; Juan et al., 2008; Masood et al., 2015; Milhome et al., 2014; Varga et al., 2013; Basaran and Ozcan, 2009). Moreover, research has explored how various factors influence AF production within a consistent nut matrix, as well as how specific nut varieties respond to fungal growth, for instance through differences in fatty acid composition (Zubair et al., 2011; Yu et al., 2004; Baazeem et al., 2021; Valente et al., 2020). Only limited information is available on how different nut matrices affect the growth of *A. flavus* and the associated production of AF. To date, only Iqbal et al. (2006) have inoculated different nuts (walnuts, peanuts, and almonds), with the primary focus of their study being on changes in nut composition rather than on a detailed characterisation of fungal growth and mycotoxin formation.

As mentioned before, the *Aspergillus* strains producing AFs, such as *A. flavus and A. parasiticus*, are predominantly native to tropical and subtropical regions with warmer temperatures (Yohannis et al., 2025; Norlia et al., 2019). However, the climate is changing, with increasing average temperatures, altered precipitation patterns, and rising atmospheric CO_2_ levels, which, in some cases, influence fungal growth; in others, mycotoxin production; and, in certain situations, both. The EFSA has already identified climate-change-driven shifts in mycotoxin production in cereals as an emerging risk, and the southern EU countries in particular are expected to be a hotspot of adverse impacts, with extreme changes in rainfall and drought, elevated temperatures, and higher CO_2_ levels affecting AF contamination in maize (Battilani et al., 2012, 2016). Although the EFSA assessment mainly focused on maize rather than nuts, this scenario remains plausible. Guo et al. (2026) have already demonstrated increased AF concentrations in Chinese peanuts attributable to climate change, and Baazeem (2021) showed that AFB1 production was significantly enhanced under specific climate change-related scenarios. It is therefore reasonable to assume that climate change will also negatively affect AF formation in nuts.

The scope of this study, therefore, was to investigate what happens if, due to climate change, temperatures increase in Germany, *A. flavus* spreads, and encounters the walnut tree native to Germany. We examined what is possible in a worst-case scenario and how the walnut compares with other nuts known to be associated with AFs.

Therefore, preliminary experiments were conducted in which German walnuts were inoculated with five different *A. flavus* strains, followed by the quantification of AFB1, B2, G1, and G2, and STC. Subsequently, different nut matrices were inoculated with two different *A. flavus* strains, which are well-known AF producers, namely AF70 and CBS119.62, and toxin production was likewise quantified. AFs and STC were analysed using an ultra-high-performance liquid chromatography method coupled with tandem mass spectrometry (UHPLC-MS/MS) after sample clean-up employing the QuEChERS (quick, easy, cheap, effective, rugged, and safe) approach combined with dispersive solid-phase extraction. The analytical method was originally developed by Dick et al. (2024) and later expanded by Schneidemann-Bostelmann et al. (2026).

## Materials and methods

### Chemicals and reagents

LC-MS grade acetonitrile (ACN) and methanol (MeOH) were sourced from Honeywell Riedel-de Haën (Seelze, Germany). LC-MS grade ultrapure water was supplied by Th. Geyer (Renningen, Germany). Sodium chloride (NaCl) was obtained from Avantor (former VWR Darmstadt, Germany), and anhydrous magnesium sulfate (MgSO₄), formic acid (FA), ammonia solution (NH₄OH), and ammonium formate solution (NH₄HCO₂) were purchased from Sigma-Aldrich (Steinheim, Germany) in analytical grade or higher. The Dispersive Solid Phase Extraction (dSPE) cleanup tubes used were Supel™ QuE PSA (primary-secondary amine)/C18 tubes containing 400 mg Discovery™ DSC-18, 1200 mg anhydrous MgSO₄, and 400 mg Supelclean™ PSA, obtained from Sigma-Aldrich (Steinheim, Germany). Oxoid™ Ringer’s solution tablets were purchased from Thermo Fisher Scientific (Darmstadt, Germany), Tween^®^ 80 from Sigma-Aldrich Canada Co (Ontario, Canada), Malt extract agar from Millipore (Ontario, Canada), and ethanol was obtained from Honeywell Riedel-de Haën (Seelze, Germany).

### Samples

Shelled but otherwise untreated walnut, cashew, hazelnut, peanut, and pistachio samples were purchased on the German market and stored at room temperature in the dark until further processing.

### Analytical standards

A mixed AF solution in acetonitrile (AFG1, AFG2, AFB1, and AFB2 at 1 µg/mL each) and unlabeled sterigmatocystin (STC) was purchased from Romer Labs (formerly Biopure, Tulln, Austria). The labeled standards [¹³C₁₇]AFG1, [¹³C₁₇]AFG2, [¹³C₁₇]AFB1, [¹³C₁₇]AFB2, and [¹³C₁₈]STC were supplied by Fianovis (formerly Libios, Vindry-sur-Turdine, France).

### Preparation of stock solutions

Purchased stock solutions of both labeled and unlabeled toxins were further diluted with acetonitrile to obtain working concentrations of 0.1, 0.01, and 0.001 µg/mL. All solutions were stored at -28 °C.

### A. flavus strains

To account for strain-specific variations, five *A. flavus* strains with differing or uncharacterized AF-producing potentials namely DSM 1959, NRRL 3357, AF 70, CBS 119.62, and NRRL 1957 were selected based on institutional availability. All *A. flavus* strains were plated on malt extract agar at the Chair of Microbiology, Technical University of Munich.

### Inoculation with A. flavus

#### Preparation of spore suspensions

*A. flavus* strains were cultured on malt extract agar to obtain spores, which were harvested using sterilized Ringer-Tween solution (25% (w/v) Ringer in water, 0.1% Tween (v/v)). The concentration of the resulting spore suspension was determined by plating serial dilutions on malt extract agar and counting colonies. The spore suspension was then adjusted to approximately 1*10^5^ spores/mL by dilution with Ringer-Tween solution and was verified by re-plating and colony counting. The preparation of the spore suspension was conducted according to a method adapted from the Fraunhofer Institute for Process Engineering and Packaging (IVV).

#### Surface disinfection

Surface disinfection was performed using 95% ethanol according to Amini et al. (2016). Nut kernels were immersed in an ethanol/water solution (95:5, v/v) and dried at room temperature overnight under a laminar flow hood (BDK, Sonnenbühl-Genkingen, Germany). Afterwards, the nuts were placed into sterile laboratory flasks fitted with a DURAN^®^ membrane-containing cap (pore size = 0.2 μm). The efficacy of surface disinfection was assessed in preliminary experiments. To this purpose, the microbiome from 10 g of walnuts was detached using 5 mL of Ringer-Tween solution and subsequently plated onto malt extract agar. These preliminary experiments confirmed successful surface disinfection of the walnut kernels with 95% ethanol. We chose this surface disinfection method to minimize alterations to the sample material, which may otherwise impact fungal growth and aflatoxin generation potential. In particular we wanted to avoid thermal changes such as those that would result from autoclaving.

#### Inoculation

Inoculation was conducted in duplicates. Walnut samples were inoculated with all different *A. flavus* strains, while cashew, hazelnut, peanut, and pistachio samples were inoculated only with the AF 70 and CBS 119.62 strains. Control samples were treated with sterile Ringer-Tween solution rather than a spore suspension. Inoculation conditions followed the protocols described by Assunção et al. (2015), with incubation for 20 days at 30 °C and 95% relative humidity in biological duplicates. 100 g of surface-disinfected kernels were mixed with spore suspension equivalent to 2% (w/w) of kernel mass, then incubated in a climatic chamber (Climacell EVO, MMM Group, Munich, Germany) under the specified conditions.

#### Spore inactivation

Spore inactivation was performed based on the thermal sensitivity of *A. flavus* spores (Zhang et al., 2018). Therefore, the samples were dried at 55 °C for 90 min directly after completion of the inoculation experiments to achieve complete spore inactivation.

### Grinding

**Fig. 4:**
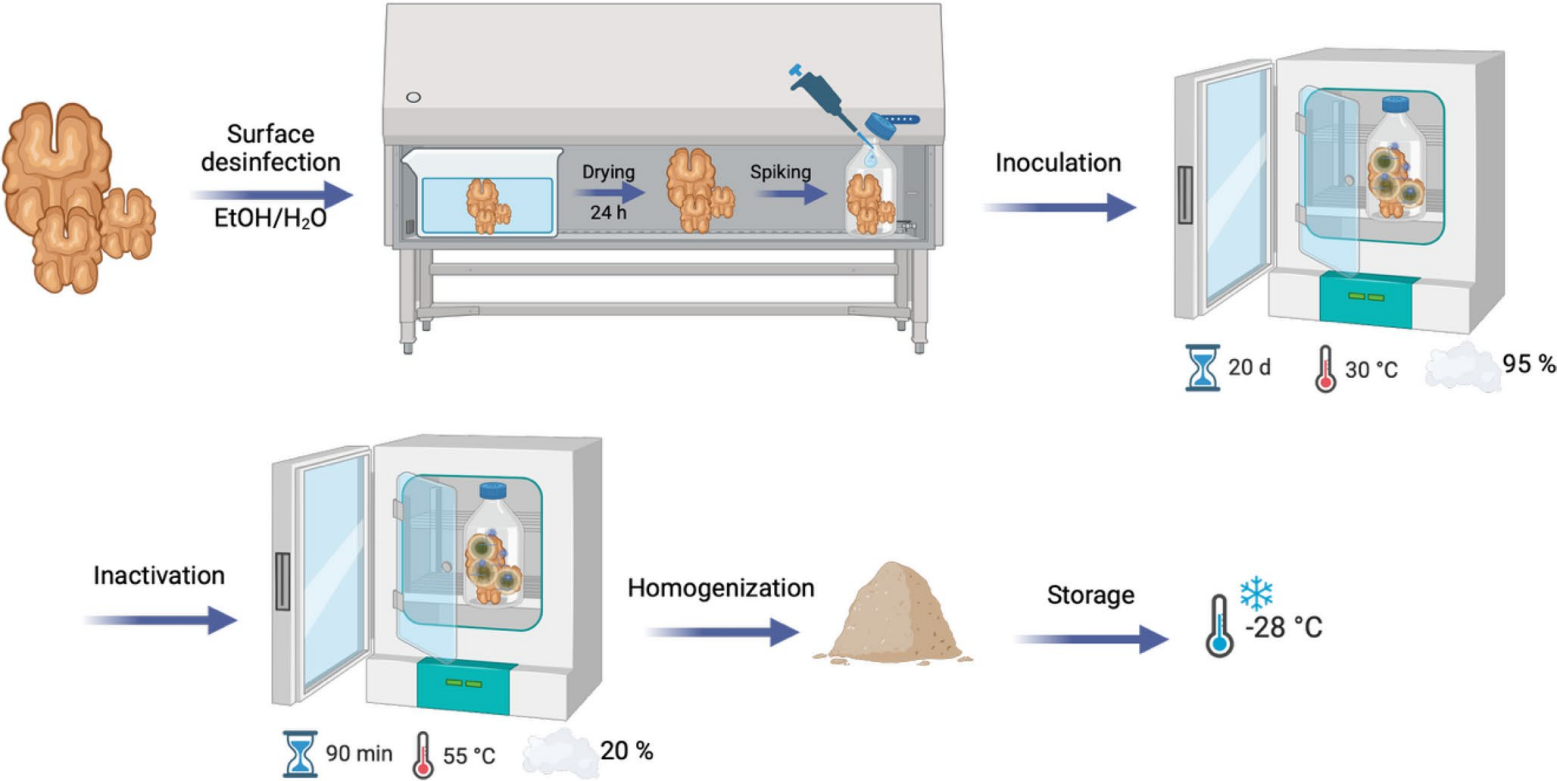
Workflow of surface disinfection and inoculation. Created in BioRender. Schneidemann, S. (2026) https://BioRender.com/lidrqkj

Each sample was subsequently ground with a laboratory mill (Grindomix GM 200, Retsch GmbH, Haan, Germany) and stored at -28 °C until further sample preparation (Fig. 4).

### Moisture content

The moisture content of the samples was determined by infrared drying using a moisture analyser (Kern & SOHN GmbH, Balingen-Frommern, Germany). For this purpose, 2.5 g of the homogenized sample were dried at 120 °C to constant weight, and the moisture content was then calculated from the recorded mass loss.

### Mycotoxin extraction

The sample preparation was performed according to the protocol of Schneidemann-Bostelmann et al. (2026) for all samples (untreated nuts, surface-disinfected nuts, control, and inoculated nuts) in triplicates. All details are summarized in the supplementary information (Supplementary Table 1).

### Validation

The applied method had already been validated according to the protocol of Vogelgesang and Hädrich (1998) (Schneidemann-Bostelmann et al. 2026), and the validation results are summarized in supplementary information (Supplementary Table 2).

### Calibration and quantitation

All analytes were quantified by means of stable isotope dilution analysis (SIDA). Calibration curves were prepared by combining a fixed amount of the stable isotope-labeled standard (IS) with different concentrations of the corresponding unlabeled analyte (A), resulting in molar ratios n(A)/n(IS) between 0.01 and 100 (1:100, 1:50, 1:10, 1:5, 1:2, 1:1, 2:1, 5:1, 10:1, 50:1, 100:1). The ratio of the chromatographic peak areas, [A(A)/A(IS)], was then plotted against the respective molar ratios [n(A)/n(IS)] to construct the calibration functions. Linear regression was applied to obtain the response curves, and their linearity was verified using the Mandel fitting test (Mandel, 1964).

### UHPLC-MS/ MS analysis

For the UHPLC-MS/MS measurements, the method previously described by Dick et al. (2024) and Schneidemann-Bostelmann et al. (2026) was used as a basis. However, all analytes except AFs and STC were removed from the method, as they were not relevant for this study. Chromatographic separation was carried out using a Shimadzu Nexera X2 UHPLC system (Shimadzu, Kyoto, Japan). A Waters BEH C18 UHPLC column (Acquity BEH C18, 100 mm, 1.7 μm × 2.1 mm; Waters GmbH, Eschborn, Germany) was employed and maintained at 40 °C. The mobile phase flow rate was set to 0.3 mL/min. The binary gradient consisted of solvent A (5 mM ammonium formate in water, adjusted to pH 9 with 25% NH₄OH solution) and solvent B (methanol). The binary gradient program began at 5% B for 2 min, followed by an increase to 18% B within 1 min. Solvent B was then raised gradually to 25% over the next 2 min. Afterwards, the proportion of B was increased to 90% within 8 min and further to 99% over 0.5 min, where it was held for 2 min. The gradient was subsequently returned to 5% B over 3.5 min, followed by a 5 min re-equilibration period. A sample volume of 10 µL was injected. To enhance peak shape, 40 µL of solvent A was co-injected in two portions of 20 µL, one before and one after the sample injection. The UHPLC system was coupled to a Shimadzu LCMS-8050 triple quadrupole mass spectrometer (Shimadzu Corporation, Kyoto, Japan). Ion source settings were as follows: interface temperature 350 °C, heat block temperature 450 °C, desolvation line temperature 150 °C, and interface voltage of + 3 kV for positive and − 3 kV for negative ionization. The heating gas and drying gas flows were each set to 10 L/min, while the nebulizing gas flow was 3 L/min. The collision-induced dissociation gas pressure was maintained at 270 kPa. Both positive and negative electrospray ionization were applied within a single analytical run by means of polarity switching. Data acquisition was performed in multiple reaction monitoring (MRM) mode. The corresponding mass transitions for all analytes are provided in Supplementary Table 3.

### Data analysis

Peak integration was performed with LabSolutions software (version 5.118; Shimadzu, Kyoto, Japan). Calibration curves, Mandel fitting tests, and analyte concentrations were calculated in Microsoft Excel for Mac (version 16.92; Microsoft, Redmond, WA, USA). The resulting quantitative data were subsequently plotted and evaluated using OriginLab 2024b (OriginLab Corporation, Northampton, MA, USA). Figure 4 was prepared using BioRender (Toronto, ON, Canada). Structural formulas were drawn using ChemDraw (version 22.0.0, Revvity Signals Software, Inc. (former PerkinElmer Informatics), Waltham, MA, USA).

### Statistical analysis

The data on AF content were statistically analysed using a t-test at a significance level of α = 0.05. Outliers were identified by applying Dixon’s Q-test (Dean and Dixon, 1951). Statistical analyses were conducted in Microsoft Excel for Mac (version 16.92; Microsoft, Redmond, WA, USA). To demonstrate potential increases in toxin concentrations during inoculation, samples containing no AFs or STC were set to their respective limits of detection (AFB1: 0.02 µg/kg; AFB2 and STC: 0.01 µg/kg), and samples with concentrations between the LOD and LOQ were set to their respective LOQ (AFB1: 0.05 µg/kg; AFB2: 0.04 µg/kg; STC: 0.02 µg/kg), which were then used for subsequent statistical analyses.

## Results and discussion

**Fig. 5:**
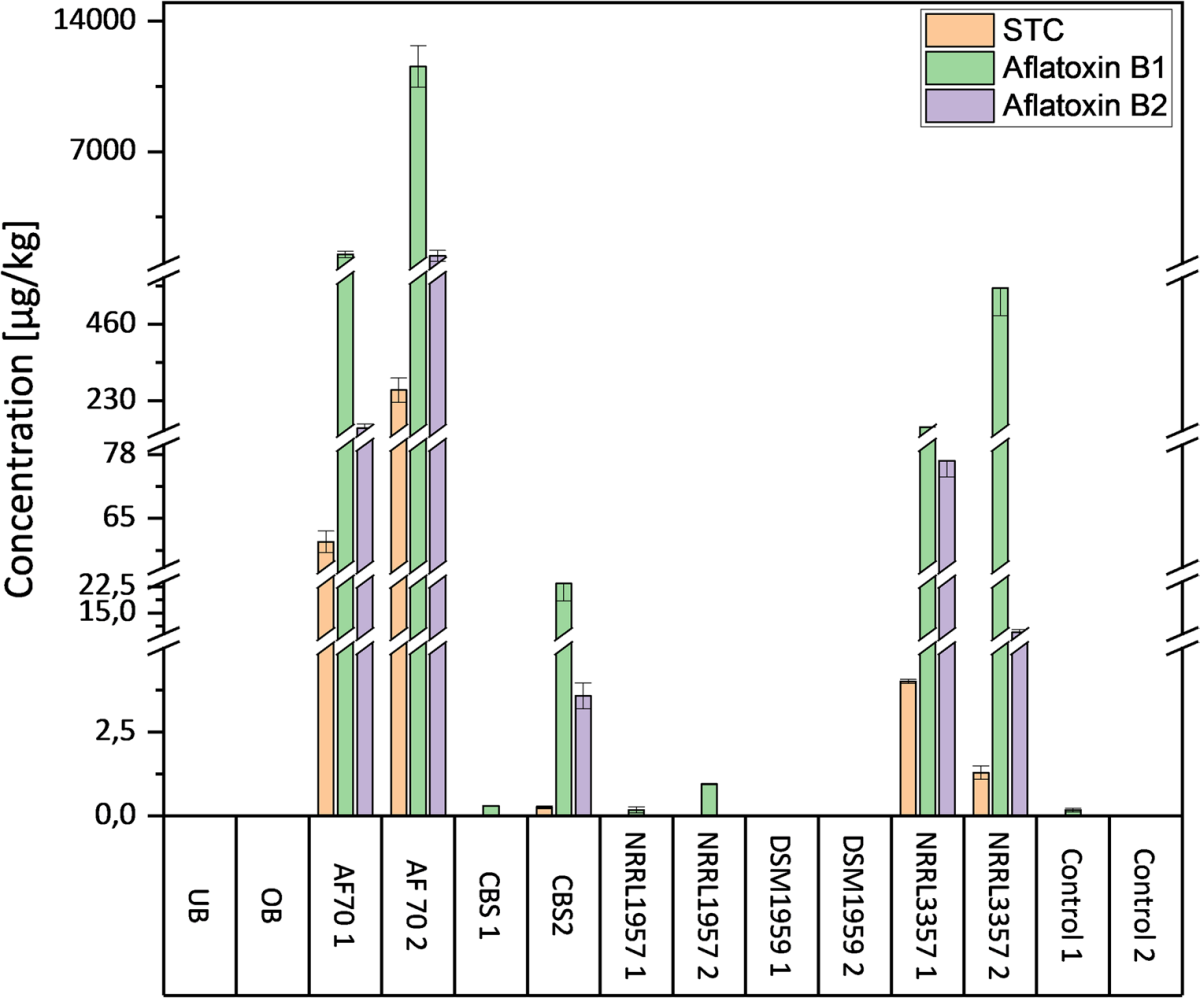
Concentration of AFB1, AFB2, and STC in walnuts, including standard deviation [µg/kg]

**Fig. 6:**
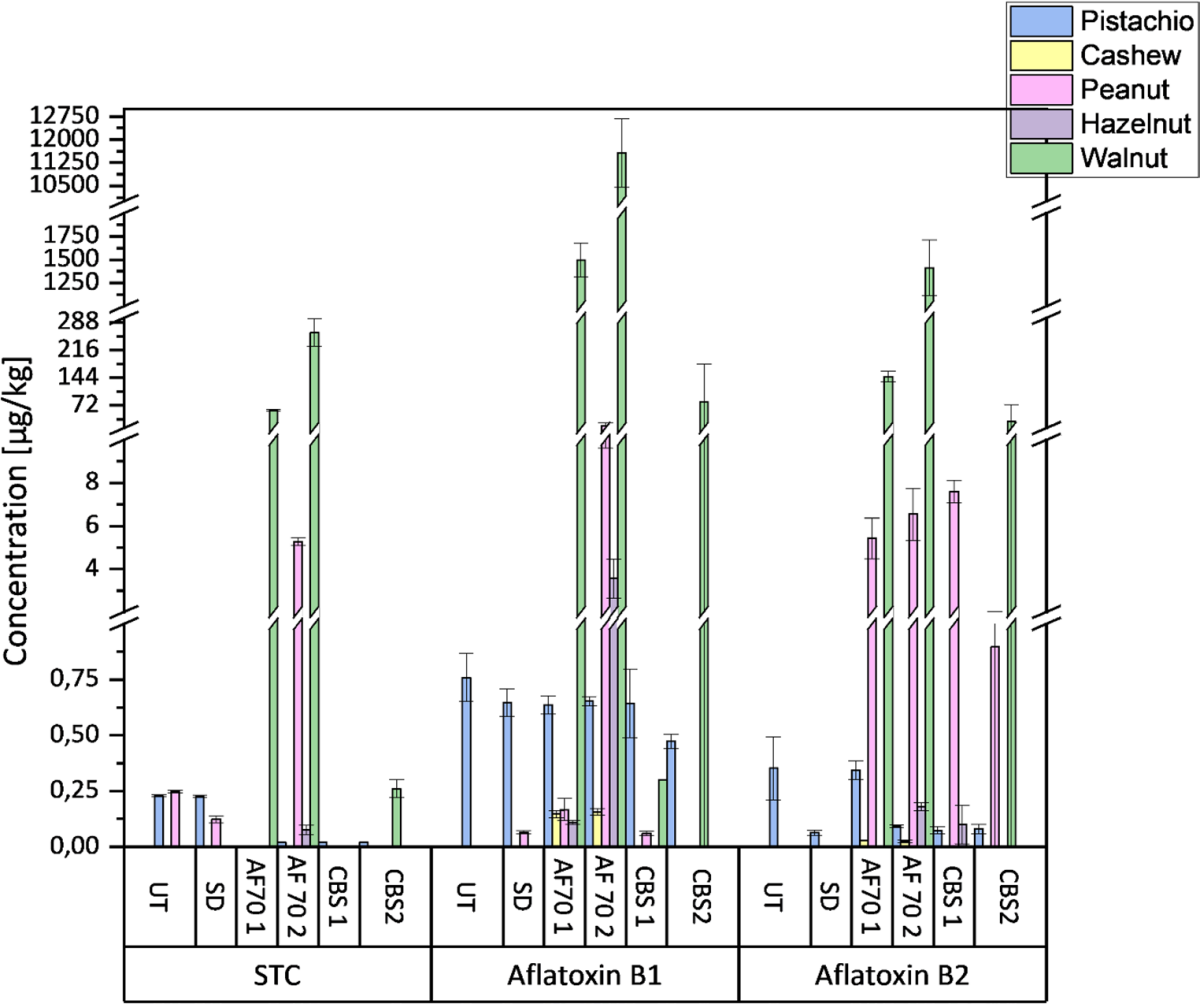
Concentration of AFB1, AFB2, and STC in different types of nuts, including standard deviation [µg/kg]

Comprehensive data on the mycotoxin levels detected in the different nut types, including AFB1, AFB2, and STC, are presented in Figs. 5 and 6. The exact concentrations are listed in the Supplementary Information (Supplementary Tables 4–7). Images of the control and nuts inoculated with *A. flavus* strains AF70 and CBS 119.62 are presented in the Supplementary Information (Supplementary Fig. 1–22).

### AF and STC production by *A. flavus* in walnuts after inoculation with different strains

 Quantification of AFs and STC in walnuts inoculated with different *A. flavus* strains revealed a strong straindependent production of AFB1, AFB2, and STC, whereas AFG1 and AFG2 were not detected in any sample. This observation aligns well with the known biosynthetic capability of *A. flavus*, which produces B-type AFs and STC but not G-type AFs (Ráduly et al., 2020). Therefore, it can be assumed that the inoculation was successful and no cross-contamination with other *Aspergillus* species occurred. The untreated walnuts showed no detectable levels of AFs or STC, nor did the surface-sterilized samples. In one of the two control samples, a low level of AFB1 (0.18 ± 0.09 µg/kg) was detected, and the sample exhibited visible fungal contamination. While the phenotypic appearance of the mycelium resembled *Aspergillus* spp., no taxonomic confirmation was performed. However, given that the walnuts originated from Germany, where natural contamination with aflatoxigenic *A. flavus* or *A. parasiticus* is ecologically highly improbable, and considering that the disinfection protocol was validated in preliminary tests, this single instance most likely represents a secondary laboratory contamination rather than a baseline colonization of the raw material.

When examining individual *A. flavus* strains, it becomes evident that almost all strains were capable of producing AFB1. Here, the reported increase in AFB1 concentrations specifically refers to the difference between the baseline levels of the initial, non-inoculated matrix and the levels detected after incubation. The AFB1 concentration increased significantly at a significance level of α = 0.05 in the samples inoculated with the AF70 strain (1494 ± 180 µg/kg and more than 11,000 µg/kg), in the samples inoculated with the CBS119.62 strain (0.30 ± 0.02 µg/kg and 23.4 ± 4.95 µg/kg), in the samples inoculated with the NRRL 1957 strain (0.18 ± 0.09 µg/kg and 0.95 ± 0.02 µg/kg), and in the samples inoculated with the NRRL 3357 strain (569 ± 82.6 µg/kg and 151 ± 1.31 µg/kg). Only the strain designated DSM 1959 was unable to produce AFB1 on walnuts. AFB2 and STC were produced at significant levels only by strains AF70 (AFB2: 147 ± 13.6 µg/kg and 1,411 ± 298 µg/kg; STC: 60.2 ± 2.17 µg/kg and 262 ± 36.1 µg/kg), NRRL 3357 (AFB2: 76.8 ± 3.92 µg/kg and 9.51 ± 0.86 µg/kg; STC: 4.00 ± 0.06 µg/kg and 1.29 ± 0.20 µg/kg), and in one biological replicate of CBS 119.62 (AFB2: 3.58 ± 0.38 µg/kg; STC: 0.26 ± 0.04 µg/kg). However, since strain NRRL 3357 exhibited markedly stronger mycelial growth than AF70 and CBS 119.62, we decided to use AF70 and CBS 119.62 for inoculating the other nut varieties. This selection was primarily a safety precaution to minimize dust formation and potential spore aerosolization during subsequent sample homogenization, thereby reducing biosafety risks. The differences observed between biological replicates may be explained by a potential hot-spot effect. If a single nut within a replicate contains a higher toxin concentration than the others, homogenization after inoculation may result in elevated total AF levels in the sample.

### AF and STC production by *A. flavus* in walnuts, hazelnuts, cashews, pistachios, and peanuts after inoculation with strains AF70 and CBS 119.62

As already observed for walnuts, only B-type AFs and STC, but no G-type AFs, were detected in cashews, pistachios, hazelnuts, and peanuts, indicating successful inoculation with *A. flavus* without cross-contamination. The untreated hazelnuts and cashews were free of AFB1, AFB2, and STC, whereas the pistachio starting material contained AFB1 (0.76 ± 0.11 µg/kg), AFB2 (0.35 ± 0.06 µg/kg), and STC (0.22 ± 0.01 µg/kg). The peanut starting material was contaminated with STC (0.25 ± 0.01 µg/kg).

When considering the two strains used for inoculation across the different nut matrices, it becomes apparent that strain AF70 was able to produce AFB1 on all nuts except pistachio, and that the AFB1 concentration increased significantly compared to the respective starting material at a significance level of α = 0.05 (Cashew: 0.11 ± 0.01 µg/kg and 0.16 ± 0.02 µg/kg; Hazelnut: 0.11 ± 0.01 µg/kg and 3.56 ± 0.90 µg/kg; Peanut: 0.16 ± 0.05 µg/kg and 17.9 ± 8.27 µg/kg). Inoculation with strain AF70 led to increased AFB2 concentrations in cashews (both samples between LOD and LOQ ), peanuts (5.43 ± 0.94 µg/kg and 6.54 ± 1.19 µg/kg), and in one biological replicate of the inoculated hazelnuts (0.18 ± 0.02 µg/kg), while STC formation was observed only in a single biological replicate of peanuts (5.28 ± 0.18 µg/kg) and hazelnuts (0.08 ± 0.02 µg/kg). On pistachios, this *A. flavus* strain did not produce any AFs or STC. In comparison with toxin formation on walnuts, it becomes evident that this strain produced markedly higher AFB1, AFB2 and STC concentrations on walnuts than on other investigated nuts.

The strain CBS119.62 was only able to produce AFB1 in one biological replicate of peanuts (0.06 ± 0.01 µg/kg) and AFB2 in peanuts (7.59 ± 1.13 µg/kg, and 0.26 ± 0.04 µg/kg) and in one biological replicate of hazelnuts (0.13 ± 0.03 µg/kg). In contrast to its behaviour on walnuts, this strain did not produce STC on any of the other investigated nuts. The *A. flavus* strain CBS 119.62 was unable to produce AFs or STC on pistachios. As described for the walnuts, the possibility of a hot-spot effect should also be considered for the other nuts, since a single contaminated kernel can result in elevated AF levels in individual samples.

### Comparison of AF and STC formation on different nut matrices and literature data

In the following, AF and STC production on the different nut matrices in this study are compared and placed in the context of published contamination levels in nuts and the reasons for AF production reported in the literature. As pointed out earlier, this study aimed to evaluate how different nut types influence *A. flavus* growth and subsequent AF production under controlled experimental conditions. To the best of our knowledge, this is the first study to systematically compare AF formation across a variety of whole, intact nut matrices, meaning that direct comparisons with identical experimental literature are not possible. Consequently, to evaluate the biological plausibility behind the distinct AF patterns and variations observed among the nut types, this discussion draws upon established literature regarding key matrix properties. Furthermore, published data on natural AF occurrence in market and stored nuts are utilized as a real-world benchmark to contextualize and validate whether the high susceptibility observed in certain nuts versus the lower accumulation in others aligns with historical surveillance data.

When comparing AF formation on the different nut matrices, it becomes apparent that the tested *A. flavus* strains produced substantially higher concentrations of B-type AFs and STC on walnuts than on the other nuts. This contrasts with the findings of Molyneux et al. (2007), who described AF-inhibiting properties of walnuts attributed to gallic acid and other antioxidant compounds. However, they also reported that these compounds must be present in the seed coat (pellicle) rather than in the endosperm, as kernel material without pellicle did not exhibit inhibitory activity, and in fact promoted AF formation at higher concentrations, presumably due to increased nutrient availability. This hypothesis is further supported by the work of Mahoney et al. (2003), who demonstrated that supplementing agar medium with walnut kernels lacking seed coats did not suppress *A. flavus* AF production; instead, higher walnut concentrations significantly enhanced it. This stimulation was attributed to either improved nutrient supply or specific kernel constituents like lipids. Conversely, incorporating walnut seed coats markedly reduced AF formation to less than 3% of control levels.

In our study, walnut kernels were purchased already shelled after industrial processing, resulting in minor injuries to the pellicle. It is therefore conceivable that fungal spores could more easily penetrate the nut through these damaged areas and, consequently, produce higher toxin levels. Tocco et al. (2009) and Adaya-Gonzales et al. (2015) further reported that cashew shells contain compounds with antimicrobial properties, the so‑called “cashew nut shell liquids” that includes anacardic acid, cardanol and cardol. These substances potentially confer resistance against *Aspergillus* colonization. Although the cashews used in our study were also obtained shelled, they showed no visible injuries, and the intact skin surrounding the kernels may have contributed to the reduced AF formation observed.

Nevertheless, the occurrence of AFs in walnuts has been repeatedly reported in the literature. Adaya-Gonzales et al. (2015) investigated walnuts, cashews, and pecans for the presence of AFs. In that study, AFB1 was detected in 35.3% of all walnut samples, making walnuts the most affected nut type investigated (for comparison: AFB1 was present in 17.7% of cashews and 12.5% of pecans). Total AF concentrations reached up to 12.1 ng/g. Adaya‑Gonzales et al. showed that, at a significance level of α = 0.05, among the nuts analysed (cashews, pecans, and walnuts), walnuts were the most heavily contaminated nuts. The importance of AFs in walnuts is further highlighted by the findings of Tokarska-Rodak et al. (2021), who identified AFs as “*the most common fungal contaminants in walnuts*”. In their study, all analysed walnut samples contained AFB1 with an average concentration of 2.5 µg/kg, and more than 65% of the samples exceeded the maximum MRL of 2 µg/kg for edible nuts. The mean concentration of total AFs was 4.8 µg/kg, with over 86% of the walnut samples surpassing the MRL of 4 µg/kg. Juan et al. (2008) likewise reported a high level of AF contamination in walnuts. In their study, peanuts, pistachios, and walnuts were analysed for AFs in general and AFB1 in particular. While only 5% of peanut samples contained detectable AFs and AFB1, 30% of walnut samples and 45% of pistachio samples were contaminated. Notably, the AF concentrations in walnuts were particularly high: the most contaminated peanut sample contained 0.17 µg/kg AFB1 and 0.30 µg/kg total AFs, whereas the most contaminated pistachio sample contained 1430 µg/kg AFB1 and 1450 µg/kg total AFs. In contrast, the most heavily contaminated walnut sample contained 2500 µg/kg AFB1 and 4320 µg/kg total AFs, with 20% of walnut samples and 5% of pistachio samples exceeding the MRL of 2 µg/kg AFB1, while no peanut sample exceeded this limit. These findings are consistent with our results, indicating that *Aspergillus* strains are indeed capable of producing high AF concentrations on walnuts.

**Fig. 7:**
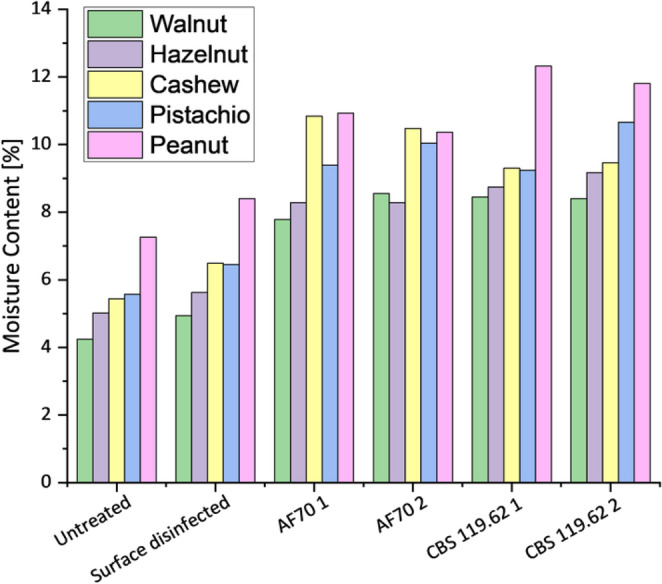
Moisture content of different types of nuts before and after the inoculation

Iqbal et al. (2006) also inoculated nuts (walnut, almond, and peanut) with *A. flavus* and monitored the increase in AF concentrations. They observed the lowest increase in peanuts and the highest in walnuts, which is consistent with our findings, in which AF levels increased in both nut types but to a much greater extent in walnuts than in peanuts. Unfortunately, this study does not report any AF concentrations or provide information on the specific *A. flavus* strain used for inoculation. Iqbal et al. hypothesized that the observed AF pattern might be due to a higher moisture content in inoculated walnuts than in peanuts. In our study, however, the inoculated peanuts had a significantly higher moisture content (at a significance level of α = 0.05) of 11.4 ± 0.87% compared with the walnuts inoculated with the same strains, which had a moisture content of 8.30 ± 0.35% (Fig. 7 and for detailed amounts Supplementary Table 8). Except for hazelnuts (8.62 ± 0.42%), all other inoculated nuts also showed significantly higher moisture contents than the corresponding inoculated walnuts (cashew: 10.0 ± 0.76%; pistachio: 9.83 ± 0.65%). Moreover, it is noteworthy that the moisture content of walnuts prior to inoculation (4.59 ± 0.49%) was significantly lower than that of the other nuts before inoculation (cashew: 5.97 ± 0.74%; pistachio: 6.01± 0.62%; peanut: 7.83 ± 0.81%; hazelnut: 5.33 ± 0.43%). Thus, our data do not support the explanation proposed by Iqbal et al. that a higher moisture content in inoculated walnuts compared with peanuts is responsible for the increased AF production in walnuts. Zubair et al. (2011) described an increase in moisture content during inoculation of walnut kernels with *A. flavus*, which they attributed to the addition of moist *A. flavus* spores. This effect was likewise observed in our experiment for all types of nuts (see Fig. 7).

Iqbal et al. (2006) demonstrated that the fat content decreased significantly during inoculation in all tested nuts. This suggests that lipids may play an important role in the growth of *Aspergillus*. However, whether this decrease in fat content is directly reflected in AF production cannot be determined from the data provided by Iqbal et al., as different amounts of AFs were produced despite significant decreases in fat content in all nut types. A decrease in crude fat content following inoculation with *A. flavus* has also been reported by Zubair et al. (2011), who inoculated whole walnuts and subsequently analysed proximate composition, fatty acid profile, and AFs. Zubair et al. further showed that, during inoculation, the walnut fatty acid profile shifted toward higher proportions of saturated fatty acids, with palmitic and stearic acids increasing while oleic, linoleic, and linolenic acids declined compared with uninoculated controls. In their correlation analysis, AFB1 concentrations were negatively associated with overall fat content and oleic acid, whereas AFB2 levels increased with rising palmitic and stearic acid contents and decreased with fat, oleic acid, and linoleic acid, suggesting that progressive depletion of unsaturated fatty acids and concomitant accumulation of certain saturated fatty acids are closely linked to AF production on walnuts. Walnuts differ from other nuts by their particularly high omega‑3 fatty acid content. In walnuts, linoleic acid accounts for almost 60% of the total lipid fraction and linolenic acid for more than 13%, whereas peanuts contain only about 4% linoleic acid and around 2% linolenic acid (for the detailed fatty acid distribution of the relevant fatty acids in each nut type, see Supplementary Table 9) (Venkatachalam and Sathe, 2006).

Xue et al. (2004) also highlighted a role of linoleate and oleate in AF formation after inoculation of peanut kernels with *A. flavus*, but in the opposite direction to what might be expected from the walnut data of Zubair et al. (2011). In their experiments, low‑linoleate lines consistently accumulated more AF, whereas lines with normal to high linoleate contents showed more variable, but overall lower, AF levels. The authors concluded that although differences in fatty acid levels explained a considerable part of the genetic variation, they were not considered reliable indicators of AF susceptibility, particularly for genotypes with oleate and linoleate contents within the normal range. Furthermore, the latter authors summarized previous work indicating that seed fatty‑acid composition can directly or indirectly affect AF production in vitro and that resistance might be enhanced by altering the fatty‑acid profile of crop seeds. Notably, supplementation of the growth medium with linoleic acid has been reported to stimulate fungal growth and/or AF formation by *Aspergillus* in some studies, while reducing it in others, underscoring the context‑dependent effect of this fatty acid on toxigenesis (Calvo et al., 1999; Doehlert et al., 1993; Fabbri et al., 1983; Passi et al., 1984). The fatty acid composition, therefore, appears to influence AF formation, but the differences observed between the individual nut matrices cannot be conclusively attributed to fatty acids alone. Moreover, the inoculation experiments in this study clearly demonstrated that AF production is highly dependent on the *A. flavus* strain used, which complicates comparisons with the literature, as none of the published studies employed the same strains as AF70 or CBS 119.62. To elucidate what specifically distinguishes walnuts from the other nut types with respect to their potential to support AFB1, AFB2, and STC formation, it would be necessary to monitor changes in fatty acid composition and other relevant constituents in each nut matrix over the course of inoculation with AF70 and CBS 119.62.

In our study, in addition to the pronounced effects observed in walnuts, we found that the *A. flavus* strain AF70 produced AFs, particularly AFB1, on cashews, hazelnuts, and peanuts, but not on pistachios. The pistachio samples, however, were already naturally contaminated with AFB1, AFB2, and STC before inoculation. According to an European Food Safety Authority (EFSA) survey (EFSA 2007) of 20,016 nut samples collected in Europe, including hazelnuts, cashews, peanuts, and pistachios (other nut types included in the EFSA survey are not considered here, as they were not part of the nut matrices investigated in our study), pistachios showed the highest frequency and levels of AF contamination among these four nut types. Samples above the limit of detection were reported for 10% of cashews, 30% of hazelnuts, 20% of peanuts and 44% of pistachios, and pistachios also had by far the highest proportion of samples with AFB1 or total AFs above 10 µg/kg (15.4% and 16.1%, respectively), compared with much lower percentages for hazelnuts (1.3%/2.9%), peanuts (2.5%/3.2%) and cashews (0.6%/0.9%). The corresponding AFB1 and total AF concentration ranges (µg/kg) likewise ranked pistachios highest (16.7–16.8 / 19.2–19.4), followed by peanuts (1.80–1.93 / 2.44–2.69), hazelnuts (0.85–0.95 / 1.50–1.70), and cashews (0.29–0.42 / 0.35–0.60). Of 168 samples exceeding 200 µg/kg, 110 were pistachios and 23 peanuts, whereas only a minor fraction were hazelnuts or cashews, confirming that, among these four nut types, pistachios are most frequently and most heavily contaminated, followed by peanuts, hazelnuts, and, lastly, cashews. A similar picture emerges from the global systematic review by Ebrahimi et al. (2022), who evaluated AF contamination in different nuts based on studies published between 2000 and 2020. In that analysis, peanuts and pistachios were the most frequently reported contaminated nut commodities, and AFB1 was the predominant AF detected in nut samples. The reported mean concentrations of total AFs and AFB1 (µg/kg), respectively, were 37.85 and 32.82 for peanuts, 31.42 and 39.44 for pistachios, 42.27 and 22.23 for walnuts, and 17.33 and 10.54 for hazelnuts, further underlining that all four nut types are relevant matrices for AF exposure, with peanuts, pistachios, and walnuts showing the highest mean contamination levels. As described earlier, a similar pattern was reported by Juan et al.(2008), who found that only 5% of peanut samples contained detectable AFs and AFB1, whereas 45% of pistachio samples were contaminated, again highlighting pistachio as the more critical commodity. In their dataset, the most contaminated peanut sample contained 0.17 µg/kg AFB1 and 0.30 µg/kg total AFs, while the most contaminated pistachio sample reached 1,430 µg/kg AFB1 and 1,450 µg/kg total AFs.

As in the EFSA survey, low AF concentrations in cashews were also observed in our study. As discussed above, one possible explanation is the presence of “cashew nut shell liquid” with antimicrobial activity, which may reduce successful colonization and toxin formation on this matrix. Consistent with literature data, we likewise found higher AF levels on hazelnuts than on cashews, but lower levels than on peanuts. Our findings also align with previous reports in showing that AF production on peanuts exceeds that on cashews and hazelnuts. Pistachios, however, do not follow this pattern. Numerous studies have reported frequent and often high AF contamination in pistachios, and the presence of AFs in the untreated pistachio samples in this work confirms that certain *Aspergillus* strains are clearly able to grow on pistachios and produce AFs. A plausible explanation is that the two strains used here, AF70 and CBS 119.62, were simply unable to synthesize AFs on the pistachio matrix under the applied conditions. To clarify this, it would be advisable to repeat the inoculation experiments on pistachios with additional *A. flavus* strains (e.g., NRRL 3357, which showed high AF production on walnuts in this study).

## Conclusion

The quantitative analysis of AFB1, AFB2, and STC in different nut-based media following inoculation with *A. flavus* revealed a pronounced strain-dependent formation of these *Aspergillus* toxins. While the tested strains differed substantially in their ability to produce AFs and STC, walnuts consistently supported distinctly higher AF formation than in cashews, hazelnuts, pistachios, and peanuts. These findings identify walnuts as a particularly favorable substrate for *A. flavus* toxin synthesis under the experimental conditions applied in this study. The mechanisms underlying this enhanced susceptibility remain unclear but may be related to walnut-specific matrix properties, such as the distinct fatty acid composition of walnuts, nutrient availability, or the integrity of the pellicle and its associated antimicrobial compounds. The observed matrix-specific differences demonstrate that the type of nut can strongly influence the toxigenic potential of *A. flavus*.

Although walnuts are currently less frequently contaminated with AFs than, for example, pistachios or peanuts, literature data indicate that, in some cases, very high AF levels can occur in this nut, such as concentrations of up to 2,500 µg/kg reported in walnuts from Morocco (Juan et al., 2008). This supports our results and shows that, although AF in walnuts are currently not concerning, the potential for walnuts to accumulate high AF concentrations may become a future health concern in Europe in the context of climate change. If climatic conditions in Europe, including Germany, change in a way that favours *Aspergillus* growth and AF formation, as projected by EFSA for other crops, this could translate into an acute health risk due to AF in native walnuts. This susceptibility may therefore become increasingly relevant for future food-safety risk assessments.

Future studies should therefore focus on inoculating intact walnut kernels to determine whether the nuts’ pellicle indeed prevents or reduces AF formation, and on deliberately damaging the pellicles of cashews and other nuts to assess the impact of seed coat integrity on toxin production. If walnuts still show outstanding AF production under these conditions, further work will be required to identify the specific walnut constituents responsible for this effect.

## Supplementary material

Below is the link to the electronic supplementary material.

Supplementary Material 1

## Data Availability

Data are provided within the manuscript and the Supplementary Information files. Raw data are available from the corresponding author upon reasonable request.

## Abbreviations

A: Aspergillus
AF: Aflatoxin
EFSA: European food safety authority
LOD: Limit of detection
LOQ: Limit of quantitation
MRL: Maximum residue level
STC: Sterigmatocystin

## References

[CR1] Abrar M, Anjum FM, Butt MS, Pasha, I. Randhawa, Saeed MA, F., Waqas K (2013) Aflatoxins: biosynthesis, occurrence, toxicity, and remedies. Crit Rev Food Sci Nutr 53:862–874 23768148 10.1080/10408398.2011.563154

[CR2] Adaya-González J, Carvajal-Moreno M, Rojo-Callejas F, Ruiz-Velasco S (2015) Aflatoxins in walnut (Juglans regia L.), pecan (Carya illinoinensis (Wangenh.) K. Koch) and cashew (Anacardium occidentale L.) nuts of Mexico. Pharm Anal Acta 6:338–350

[CR3] Amini M, Ghoranneviss M (2016) Effects of cold plasma treatment on antioxidants activity, phenolic contents and shelf life of fresh and dried walnut (Juglans regia L.) cultivars during storage. Lwt 73:178–184

[CR4] Assuncao E, Reis TA, Baquiao AC, Correa B (2015) Effects of gamma and electron beam radiation on Brazil nuts artificially inoculated with Aspergillus flavus. J Food Prot 78:1397–1401 26197295 10.4315/0362-028X.JFP-14-595

[CR5] Baazeem A, Rodriguez A, Medina A, Magan N (2021) Impacts of climate change interacting abiotic factors on growth, aflD and aflR gene expression and aflatoxin B1 production by Aspergillus flavus strains in vitro and on pistachio nuts. Toxins 13:385 34071166 10.3390/toxins13060385 PMC8228473

[CR6] Basaran P, Ozcan M (2009) Occurrence of aflatoxins in various nuts commercialized in Turkey. J Food Saf 29:95–105

[CR7] Battilani P, Rossi V, Giorni P, Pietri A, Gualla A, Van der Fels‐Klerx HJ, Booij CJ, Moretti A, Logrieco A, Miglietta F, Toscano P (2012) Modelling, predicting and mapping the emergence of aflatoxins in cereals in the EU due to climate change. EFSA Supporting Publications 9:223E

[CR8] Battilani P, Toscano P, Van Der Fels-Klerx H, Camardo Leggieri Morettia, Goumperis Mbreracrortaisa T, Robinson T (2016) Aflatoxin B1 contamination in maize in Europe increases due to climate change. Sci Rep 6:24328 27066906 10.1038/srep24328 PMC4828719

[CR9] Bayman P, Baker, J. L., Mahoney NE (2002) Aspergillus on tree nuts: incidence and associations. Mycopathologia 155:161–169 12617503 10.1023/a:1020419226146

[CR10] Bundesanstalt FüR Landwirtschaft Und Ernährung (2025) Konsum von Nüssen und Schalenobst in Deutschland in den Jahren 2010/11 bis 2024/25 (in 1.000 Tonnen) [Online]. Statista: BMEL Statistik. Available: https://de.statista.com/statistik/daten/studie/1090168/umfrage/verbrauch-von-nuessen-und-schalenobst-in-deutschland/

[CR11] BVL (2013) Monitoring 2013. In: Lebensmittelsicherheit BFVU (ed) BVL-Report, 9.3, pp 1–104

[CR12] BVL (2019) Monitoring 2019. In: Lebensmittelsicherheit BFVU (ed) *Berichte zur Lebensmittelsicherheit*. BVL-Report, 15.3, pp 1–163

[CR13] Calvo AM, Hinze LL, Gardner, H. W., Keller NP (1999) Sporogenic effect of polyunsaturated fatty acids on development of Aspergillus spp. Appl Environ Microbiol 65:3668–3673 10427064 10.1128/aem.65.8.3668-3673.1999 PMC91549

[CR14] Cunha SC, SA SV, Fernandes JO (2018) Multiple mycotoxin analysis in nut products: Occurrence and risk characterization. Food Chem Toxicol 114:260–269 29458161 10.1016/j.fct.2018.02.039

[CR15] Dean RB, Dixon WJ (1951) Simplified statistics for small numbers of observations. Anal Chem 23:636–638

[CR16] Dick F, Dietz A, Asam S, Rychlik M (2024) Development of a high-throughput UHPLC-MS/MS method for the analysis of Fusarium and Alternaria toxins in cereals and cereal-based food. Anal Bioanal Chem 416:5619–5637 39222085 10.1007/s00216-024-05486-4 PMC11493838

[CR17] Doehlert DC, Wicklow DT, Gardner HW (1993) Evidence implicating the lipoxygenase pathway in providing resistance to soybeans against Aspergillus flavus. Phytopathology 83:1473–1477

[CR18] Ebrahimi A, Emadi A, Arabameri M, Jayedi A, Abdolshahi A, YANCHESHMEH BS, Shariatifar N (2022) The prevalence of aflatoxins in different nut samples: A global systematic review and probabilistic risk assessment. AIMS Agric Food 7(1):130–148

[CR19] EFSA (2007) Opinion of the scientific panel on contaminants in the food chain [CONTAM] related to the potential increase of consumer health risk by a possible increase of the existing maximum levels for aflatoxins in almonds, hazelnuts and pistachios and derived products. EFSA J 5:446

[CR20] Efsa, Schrenk D, Bignami M, Bodin L, Del Chipmanjk, Mazo J, Grasl-Kraupp B, Hogstrand, C., Hoogenboom, L. R., Leblanc J-C (2020) Risk assessment of aflatoxins in food. 10.2903/j.efsa.2020.6040 PMC7447885 32874256

[CR22] European commission (2024) 2023 Annual Report Alert and Cooperation Network. Publications Office of the European Union Luxembourg

[CR23] European commission (2025) 2024 Annual Report Alert and Cooperation Network. Publications Office of the European Union Luxembourg

[CR21] European commission (2023) Commission Regulation (EU) 2023/915 of 25 April 2023 on maximum levels for certain contaminants in food and repealing Regulation (EC) No 1881/2006. In: UNION E (ed) Official Journal of the European Union

[CR24] Fabbri AA, Fanelli C, Panfili G, Passi S, Fasella P (1983) Lipoperoxidation and aflatoxin biosynthesis by Aspergillus parasiticus and A. flavus. Microbiology 129:3447–3452

[CR25] Gao W, Jiang L, GE L, Chen M, Yang Gengc, LI G, JI Q, F., Yan, Q., Zou Y (2015) Sterigmatocystin-induced oxidative DNA damage in human liver-derived cell line through lysosomal damage. Toxicol In Vitro 29:1–7 25176419 10.1016/j.tiv.2014.08.007

[CR26] Guo C, Zhao Y, Liu A, Wang D, Wang X, Yu L, Ma F, Wang X, Fang M, Ding X, Logrieco AF (2026) Dynamic changes and early warning of peanuts aflatoxin B1 contamination in China in the context of climate change. npj Sci Food 10:47 41559122 10.1038/s41538-025-00696-1 PMC12891717

[CR27] Habibipour R, Tamandegani, P. R., Farmany A (2016) Monitoring of aflatoxin G1, B1, G2, and B2 occurrence in some samples of walnut. Environ Monit Assess 188:669 27848109 10.1007/s10661-016-5678-4

[CR28] Iarc Wgoteocrth (2012) Chemical agents and related occupations. IARC Monogr Eval Carcinog Risks Hum 100:9 23189753 PMC4781612

[CR29] International agency for research on cancer (1987) Some naturally occurring substances. IARC Monogr Eval Carcinog Risks Hum, vol 1–42, p 449

[CR30] Iqbal A, Khalil IA, Shah H (2006) Aflatoxin contents of stored and artificially inoculated cereals and nuts. Food Chem 98:699–703

[CR31] Juan C, Idrissi Zinedineamoltoj, L., Manes J (2008) Aflatoxins levels in dried fruits and nuts from Rabat-Salé area, Morocco. Food Control 19:849–853

[CR32] Liu Y, DU M, Zhang G (2014) Proapoptotic activity of aflatoxin B1 and sterigmatocystin in HepG2 cells. Toxicol Rep 1:1076–1086 28962319 10.1016/j.toxrep.2014.10.016 PMC5598229

[CR33] Mahoney N, Leslie Molyneuxrmckennaj, C., Mcgranahan G (2003) Resistance of ‘Tulare’walnut (Juglans regia cv. Tulare) to aflatoxigenesis. J Food Sci 68:619–621

[CR34] Mandel J (1964) The statistical analysis of experimental data. Interscience Publishers, J. Wiley & Sons, New York

[CR35] Masood M, Asi Iqbalsz, M. R., Malik N (2015) Natural occurrence of aflatoxins in dry fruits and edible nuts. Food Control 55:62–65

[CR36] Milhome M, DE Limac, Lima L, Lima F, Sousa D, Nascimento R (2014) Occurrence of aflatoxins in cashew nuts produced in northeastern Brazil. Food Control 42:34–37

[CR37] Molyneux RJ, Mahoney, N., Kim, J. H., Campbell BC (2007) Mycotoxins in edible tree nuts. Int J Food Microbiol 119:72–78 17719114 10.1016/j.ijfoodmicro.2007.07.028

[CR38] Nakai VK, DE Oliveira Rocha L, Gonçalez E, Fonseca H, Ortega EMM, Corrêa B (2008) Distribution of fungi and aflatoxins in a stored peanut variety. Food Chem 106:285–290

[CR39] Norlia M, Nor-Khaizura Jinaps, Radu Mar NI, Ssamsudin P, Azri FA (2019) Aspergillus section Flavi and aflatoxins: Occurrence, detection, and identification in raw peanuts and peanut-based products along the supply chain. Front Microbiol 10:2602 31824445 10.3389/fmicb.2019.02602 PMC6886384

[CR40] Passi S, Nazzaro-Porro M, Fabbri Fanellic A, Fasella P (1984) Role of lipoperoxidation in aflatoxin production. Appl Microbiol Biotechnol 19:186–190

[CR41] Ráduly Z, Szabó L, Pócsi Madara, I., Csernoch L (2020) Toxicological and medical aspects of Aspergillus-derived mycotoxins entering the feed and food chain. Front Microbiol 10:2908 31998250 10.3389/fmicb.2019.02908 PMC6962185

[CR42] Rank C, Nielsen KF, Larsen TO, Varga J, Samson RA, Frisvad JC (2011) Distribution of sterigmatocystin in filamentous fungi. Fungal Biology 115:406–420 21530923 10.1016/j.funbio.2011.02.013

[CR43] Schneidemann-Bostelmann S, Otting Y, Dick F, Lefever T, Asam S, Rychlik M (2026) Mycotoxin occurrence and risk assessment in plant-based meat, cheese, and fish alternatives based on an adapted UHPLC-MS/MS multi-method. Mycotoxin Res 42:22 41632390 10.1007/s12550-026-00636-2 PMC12868108

[CR44] Škrbić B, Živančev J, Godula M (2014) Multimycotoxin analysis of crude extracts of nuts with ultra-high performance liquid chromatography/tandem mass spectrometry. J Food Compos Anal 34:171–177

[CR46] Statista (2025b) Umsatz mit Nüssen weltweit nach Ländern im Jahr 2024 (in Millionen Euro) [Online]. Statista: Statista Market Insights. Available: https://de.statista.com/statistik/daten/studie/1349402/umfrage/umsatz-mit-nuessen-weltweit-nach-laendern/

[CR45] Statista (2025a) Umsatz mit Nüssen in Deutschland in den Jahren 2024 bis 2030 (in Millionen Euro) [Online]. Statista: Statista Market Insights. Available: https://de.statista.com/statistik/daten/studie/1104497/umfrage/umsatz-mit-nuessen-und-nussmischungen/

[CR47] Statistisches Bundesamt (2025) Landwirtschaftliche Nutzfläche zum Anbau von Nüssen in Deutschland in den Jahren 2010 bis 2024 (in Hektar) [Online]. Statista: Statistisches Bundesamt. Available: https://de.statista.com/statistik/daten/studie/1102500/umfrage/anbauflaeche-von-nuessen-in-deutschland/

[CR48] Tocco G, Meli Faisa, Begala G, Podda M, Fadda G, Filippone Mbcordamattanasioa, P., Berretta S (2009) PEG-immobilization of cardol and soluble polymer-supported synthesis of some cardol–coumarin derivatives: Preliminary evaluation of their inhibitory activity on mushroom tyrosinase. Bioorg Med Chem Lett 19:36–39 19054671 10.1016/j.bmcl.2008.11.020

[CR49] Tokarska-Rodak M, Zarębska M, Nitychoruk DK (2021) Mycotoxin Contaminants of Walnuts-Preliminary Studies. Pol J Environ Stud 31:493–498

[CR50] Turner NW, Subrahmanyam, S., Piletsky SA (2009) Analytical methods for determination of mycotoxins: a review. Anal Chim Acta 632:168–180 19110091 10.1016/j.aca.2008.11.010

[CR51] Valente S, Prencipe Melonigr, Spigolon S, Fontana Nsomenzim, Gullino M, M. L., Spadaro D (2020) Effect of drying temperatures and exposure times on Aspergillus flavus growth and aflatoxin production on artificially inoculated hazelnuts. J Food Prot 83:1241–1247 32221534 10.4315/JFP-20-061

[CR52] Varga E, Glauner T, Berthiller F, Krska R, Schuhmacher R, & Sulyok M (2013) Development and validation of a (semi-) quantitative UHPLC-MS/MS method for the determination of 191 mycotoxins and other fungal metabolites in almonds, hazelnuts, peanuts and pistachios. Anal Bioanal Chem 405:5087–5104 23471368 10.1007/s00216-013-6831-3 PMC3656230

[CR53] Venkatachalam M, Sathe SK (2006) Chemical composition of selected edible nut seeds. J Agric Food Chem 54:4705–4714 16787018 10.1021/jf0606959

[CR54] Vogelgesang J, Hädrich J (1998) Limits of detection, identification and determination: a statistical approach for practitioners. Accred Qual Assur 3:242–255

[CR55] Yohannis E, Urugo MM, Getachew Tekata, Tola P, Kebede Ybforsidosf, Y. S., Teferra TF (2025) Aflatoxin Contamination in Agri-Food Systems: A Comprehensive Review of Toxicity, Food Security, Economic Impacts, and Sustainable Mitigation Across the Value Chain. Food Sci Nutr 13:e71104 41111890 10.1002/fsn3.71104 PMC12529245

[CR56] Yu J, Bhatnagar Changp-Kehrlichkccaryjw, Cleveland D, Payne TE, G. A., Linz, J. E., Woloshuk, C. P., Bennett JW (2004) Clustered pathway genes in aflatoxin biosynthesis. Appl Environ Microbiol 70:1253–1262 15006741 10.1128/AEM.70.3.1253-1262.2004 PMC368384

[CR57] Zhang S, Zhang L, Lan R, Zhou X, Kou X, Wang S (2018) Thermal inactivation of Aspergillus flavus in peanut kernels as influenced by temperature, water activity and heating rate. Food Microbiol 76:237–244 30166147 10.1016/j.fm.2018.05.015

[CR58] Zubair A, Zahoor U, Hamid Shera, Barkat U, A., Ali E (2011) Fatty acid profile and aflatoxin contamination of walnuts (Juglans regia). ARPN J Agricultural Biol Sci 6:1–7

