# Supplementary material for: Quantification of aflatoxins and sterigmatocystin in walnuts, cashews, pistachios, peanuts, and hazelnuts by using UHPLC-MS/MS following Aspergillus flavus inoculation
Source: Mycotoxin Res. 2026 Aug 4;42(3):53. doi: 10.1007/s12550-026-00666-w (PMC13437669; doi:10.1007/s12550-026-00666-w)
Supplement: Supplementary file 1 [file 12550_2026_666_MOESM1_ESM.pdf]

Supplementary Information

Supplementary table 1: Sample preparation.

Supplementary table 2: Results of the method validation*, including LOD, LOQ, relative standard deviation (RSD), and recovery, validated in a blank matrix*.

Supplementary table 3: LC-MS/MS parameters of the analysed mycotoxins.

Supplementary table 4: Concentrations of STC and Aflatoxins in walnuts, including standard deviation [µg/kg]. Mycelial growth as assessed visually.

Supplementary table 5: Concentrations of AFB1 in different types of nuts, including standard deviation [µg/kg].

Supplementary table 6: Concentrations of AFB2 in different types of nuts, including standard deviation [µg/kg].

Supplementary table 7: Concentrations of STC in different types of nuts, including standard deviation [µg/kg].

Supplementary table 8: Moisture content of different types of nuts before and after the inoculation, including standard deviation [%].

Supplementary table 9: Distribution of the relevant fatty acids in each nut type. All values are expressed as grams per 100 g of lipid (Venkatachalam and Sathe, 2006).

Supplementary table 1: Sample preparation.

Step	Description
1: Sample Preparation	Weigh 1 g of homogenized, freeze-dried sample into a 15 mL centrifuge tube and spike with isotope-labeled internal standards. Evaporate solvent overnight under a fume hood.
2: Extraction	Add 10 mL ACN/H_2O (80/20, v/v) and 100 µL formic acid (FA). Shake at 400 rpm for 60 min using a horizontal shaker (Kombischüttler KL 2, Edmund Bühler GmbH, Hechingen, Germany).
3: Centrifugation	Centrifuge at 3,220 g for 5 min (Centrifuge 5810 R, Eppendorf AG, Hamburg, Germany). Transfer supernatant to a clean 50-mL centrifuge tube.
4: Extraction	Re-extract residue with 5 mL ACN/H_2O (80/20, v/v) + 50 µL FA for 30 min. Centrifuge (3,220 g, 5 min).
5: Extraction	Extract again with 5 mL ACN/H_2O (70/30, v/v) + 50 µL FA for 30 min. Centrifuge (3,220 g, 5 min).
6: Acidification & QuEChERS clean-up	Combine supernatants, acidify with 200 µL FA, add 1.8 g $MgSO_4$ and 0.45 g NaCl, vortex 1 min (QuEChERS cleanup).
7: d-SPE cleanup	Centrifuge (3,220 g, 5 min) and transfer 8 mL ACN phase to d-SPE tube. Shake 15 min and centrifuge again.
8: Evaporation	Transfer supernatant to 4 mL glass vial and evaporate to dryness under nitrogen at 40 °C in two steps (Evaporator EVA-EC2-48-S, Zefa-Laborservice, Harthausen).
9: Reconstitution	Reconstitute residue in 200 µL MeOH/H_2O (60/40, v/v) and freeze ≥30 min at -28 °C.
10: Completion	Filter (PVDF, 0.2 µm), centrifuge 15 min at 13,201 g (Laborzentrifuge 2 K15, Sigma, Osterode am Harz, Germany) and 4 °C, transfer to glass vial with micro-insert, and store at -28 °C.

Supplementary table *2*: Results of the method validation, including LOD, LOQ, relative standard deviation (RSD), and recovery, validated in a blank matrix.

Analyte	LOD [µg/kg]	LOQ [µg/kg]	Precision (RSD) [%]			Recovery [%]			
			Inter-injection (n=10)	Intra-day (n=3)	Inter-day (n=9)	Level 1	Level 2	Level 3	Level 4
AFB1	0.02	0.05	3.00	8.26	7.07	116	86.4	89.4	114
AFB2	0.01	0.04	2.05	7.00	7.73	96.3	95.3	90.8	98.6
AFG1	0.02	0.07	2.04	3.04	8.28	96.3	99.8	100	103
AFG2	0.28	0.84	3.68	8.99	5.69	97.4	92.1	96.7	98.7
STC	0.01	0.02	3.47	2.14	9.20	119	105	98.9	75.9

Supplementary table 3: LC-MS/MS parameters of the analysed mycotoxins.

Analyte	Measured ion	Precursor ion (m/z)	Product ions (m/z)	Q1 Pre bias (V)	Collision energy [V]	Q3 Pre bias (V)	Retention time (min)
				Qualifier/Quantifier			
AFG2	$[M + H]^+$	331.20	189.10/115.20	-18.0/-14.0	-42.0/-65.0	-20.0/-24.0	8.98
$[^{13}C_{17}]$- AFG2	$[M + H]^+$	348.20	199.10/123.20	-18.0/-14.0	-42.0/-65.0	-20.0/-24.0	8.98
AFG1	$[M + H]^+$	329.00	243.15/200.10	-16.0/-14.0	-26.0/-43.0	-26.0/-22.0	9.24
$[^{13}C_{17}]$- AFG1	$[M + H]^+$	346.00	257.15/212.10	-16.0/-14.0	-26.0/-43.0	-26.0/-22.0	9.24
AFB2	$[M + H]^+$	315.00	287.15/259.10	-16.0/-16.0	-26.0/-30.0	-20.0/-18.0	9.58
$[^{13}C_{17}]$- AFB2	$[M + H]^+$	332.00	303.15/274.10	-16.0/-16.0	-26.0/-30.0	-20.0/-18.0	9.58
AFB1	$[M + H]^+$	313.00	285.15/241.10	-16.0/-16.0	-23.0/-36.0	-20.0/-26.0	9.82
$[^{13}C_{17}]$- AFB1	$[M + H]^+$	330.00	301.15/256.10	-16.0/-16.0	-23.0/-36.0	-20.0/-26.0	9.82
STC	$[M + H]^+$	324.90	310.20/281.15	-16.0/-16.0	-23.0/-36.0	-20.0/-26.0	12.3
$[^{13}C_{18}]$-STC	$[M + H]^+$	342.90	327.20/298.15	-16.0/-16.0	-23.0/-36.0	-20.0/-26.0	12.3

Supplementary table 4: Concentrations of STC and Aflatoxins in walnuts, including standard deviation [µg/kg]. Mycelial growth as assessed visually.

	Mycelial Growth	STC [µg/kg]	AFB1 [µg/kg]	AFB2 [µg/kg]
Untreated	-	-	-	-
Surface disinfected	-	-	-	-
Control 1	-	-	0.18 ± 0.05	-
Control 2	+	-	-	-
AF70 1	+	60.2 ± 2.17	1,494 ± 180	147 ± 13.6
AF702	+	262 ± 36.1	> 11,000**	1,411 ± 298
DSM1959 1	++	-	-	-
DSM1959 2	++	-	-	-
sNRRL1957 1	++	-	0.18 ± 0.09	-
NRRL1957 2	++	-	0.95 ± 0.02*	-
CBS119.62 1	+	-	0.30 ± 0.02	-
CBS119.62 2	++	0.26 ± 0.04	23.4 ± 4.95*	3.58 ± 0.38*
NRRL3357 1	++	4.00 ± 0.06	151 ± 1.31	76.8 ± 3.29
NRRL3357 2	++	1.29 ± 0.20	569 ± 82.6*	9.51 ± 0.86*

* Outliers identified via Dixon's Q test and excluded from mean calculation and statistical evaluation

** Detector signal saturated

Supplementary table 5: Concentrations of AFB1 in different types of nuts, including standard deviation [µg/kg].

	Walnut	Hazelnut	Cashew	Pistachio	Peanut
Untreated	-	-	-	0.76 ± 0.11	-
Surface disinfected	-	-	-	0.65 ± 0.06	0.06 ± 0.01
AF70 1	$1{,}494 \pm 180$	0.11 ± 0.01	0.15 ± 0.02	0.64 ± 0.04	0.16 ± 0.05
AF70 2	> 11,000**	3.56 ± 0.90	0.16 ± 0.02	0.65 ± 0.02	17.9 ± 8.27
CBS 119.62 1	0.30 ± 0.02	-	-	0.64 ± 0.15	0.06 ± 0.01
CBS 119.62 2	23.4 ± 4.95*	-	-	0.47 ± 0.03	-

* Outliers identified via Dixon's Q test and excluded from mean calculation and statistical evaluation
** Detector signal saturated

Supplementary table 6: Concentrations of AFB2 in different types of nuts , including standard deviation [µg/kg].

	Walnut [µg/kg]	Hazelnut [µg/kg]	Cashew [µg/kg]	Pistachio [µg/kg]	Peanut [µg/kg]
Untreated	-	-	-	0.35 ± 0.14	-
Surface disinfected	-	-	-	0.06 ± 0.01	-
AF70 1	147 ± 13.6	-	< LOQ	0.34 ± 0.04	5.43 ± 0.94
AF70 2	1,411 ± 298	0.18 ± 0.02	< LOQ	0.09 ± 0.01	6.54 ± 1.19
CBS 119.62 1	-	0.13 ± 0.03	-	0.07 ± 0.02	7.59 ± 1.13
CBS 119.62 2	3.58 ± 0.38*	-	-	0.08 ± 0.02	0.26 ± 0.04

* Outliers identified via Dixon's Q test and excluded from mean calculation and statistical evaluation

Supplementary table 7: Concentrations of STC in different types of nuts, including standard deviation [µg/kg].

	Walnut [µg/kg]	Hazelnut [µg/kg]	Cashew [µg/kg]	Pistachio [µg/kg]	Peanut [µg/kg]
Untreated	-	-	-	0.22 ± 0.01	0.25 ± 0.01
Surface disinfected	-	-	-	0.23 ± 0.1	0.12 ± 0.02
AF70 1	60.2 ± 2.17	-	-	-	-
AF70 2	262 ± 36.1	0.08 ± 0.02	-	LOQ	5.28 ± 0.18
CBS 119.62 1	-	-	-	LOQ	-
CBS 119.62 2	0.26 ± 0.04	-	-	LOQ	-

Supplementary table 8: Moisture content of different types of nuts before and after the inoculation, including standard deviation [%].

	Walnut	Hazelnut	Cashew	Pistachio	Peanut
Before inoculation	4.59 ± 0.49	5.33 ± 0.43	5.97 ± 0.74	6.01± 0.62	7.83 ± 0.81
After inoculation (with AF70 and CBS119.62)	8.30 ± 0.35	8.62 ± 0.42	10.0 ± 0.76	9.83 ± 0.65	11.4 ± 0.87

Supplementary table 9: Distribution of the relevant fatty acids in each nut type. All values are expressed as grams per 100 g of lipid (Venkatachalam and Sathe, 2006).

Fatty Acid	Cashew	Hazelnut	Pistachio	Walnut	Peanut
16:0 (Palmitic acid)	10.7 ± 0.02	5.78 ± 0.01	12.7 ± 0.16	8.14 ± 0.11	6.20 ± 0.01
18:0 (Stearic acid)	9.33 ± 0.03	3.12 ± 0.05	1.09 ± 0.00	2.84 ± 0.04	2.06 ± 0.01
18:1 (Oleic acid)	61.2 ± 0.03	83.0 ± 0.04	50.3 ± 0.14	15.2 ± 0.05	81.3 ± 0.06
18:2 (Linoleic acid)	16.9 ± 0.07	7.55 ± 0.02	33.4 ± 0.07	59.8 ± 0.01	3.87 ± 0.00
18:3 (Linolenic acid)	0.32 ± 0.00	0.24 ± 0.00	0.86 ± 0.00	13.2 ± 0.01	1.79 ± 0.00

Supplementary figure 1-2 Walnut control 1 (left), walnut control 2 (right)

Supplementary figure 3-4 Walnut AF70 1 (left), walnut AF70 2 (right)

Supplementary figure 5-6 Walnut CBS 119.62 1 (left), walnut CBS 119.62 2 (right)

Supplementary figure 7-8 Cashew AF70 1 (left), cashew AF70 2 (right)

Supplementary figure 9-10 Cashew CBS 119.62 1 (left), cashew CBS 119.62 2 (right)

Supplementary figure 11-12 Peanut AF70 1 (left), peanut AF70 2 (right)

Supplementary figure 13-14 Peanut CBS 119.62 1 (left), peanut CBS 119.62 2 (right)

Supplementary figure 15-16 Hazelnut AF70 1 (left), hazelnut AF70 2 (right)

Supplementary figure 17-18 Hazelnut CBS 119.62 1 (left), hazelnut CBS 119.62 2 (right)

Supplementary figure 19-20 Pistachio AF70 1 (left), pistachio AF70 2 (right)

Supplementary figure 21-22 Pistachio CBS 119.62 1 (left), pistachio CBS 119.62 2 (right)

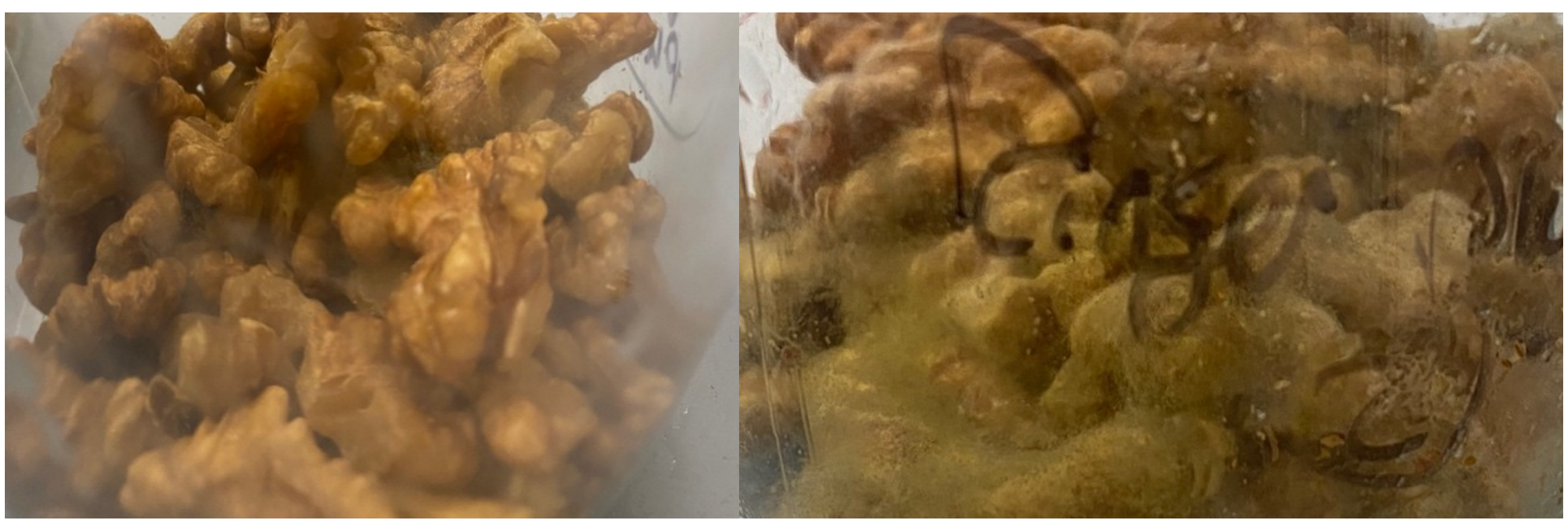

Supplementary figure 1-2 Walnut control 1 (left), walnut control 2 (right)

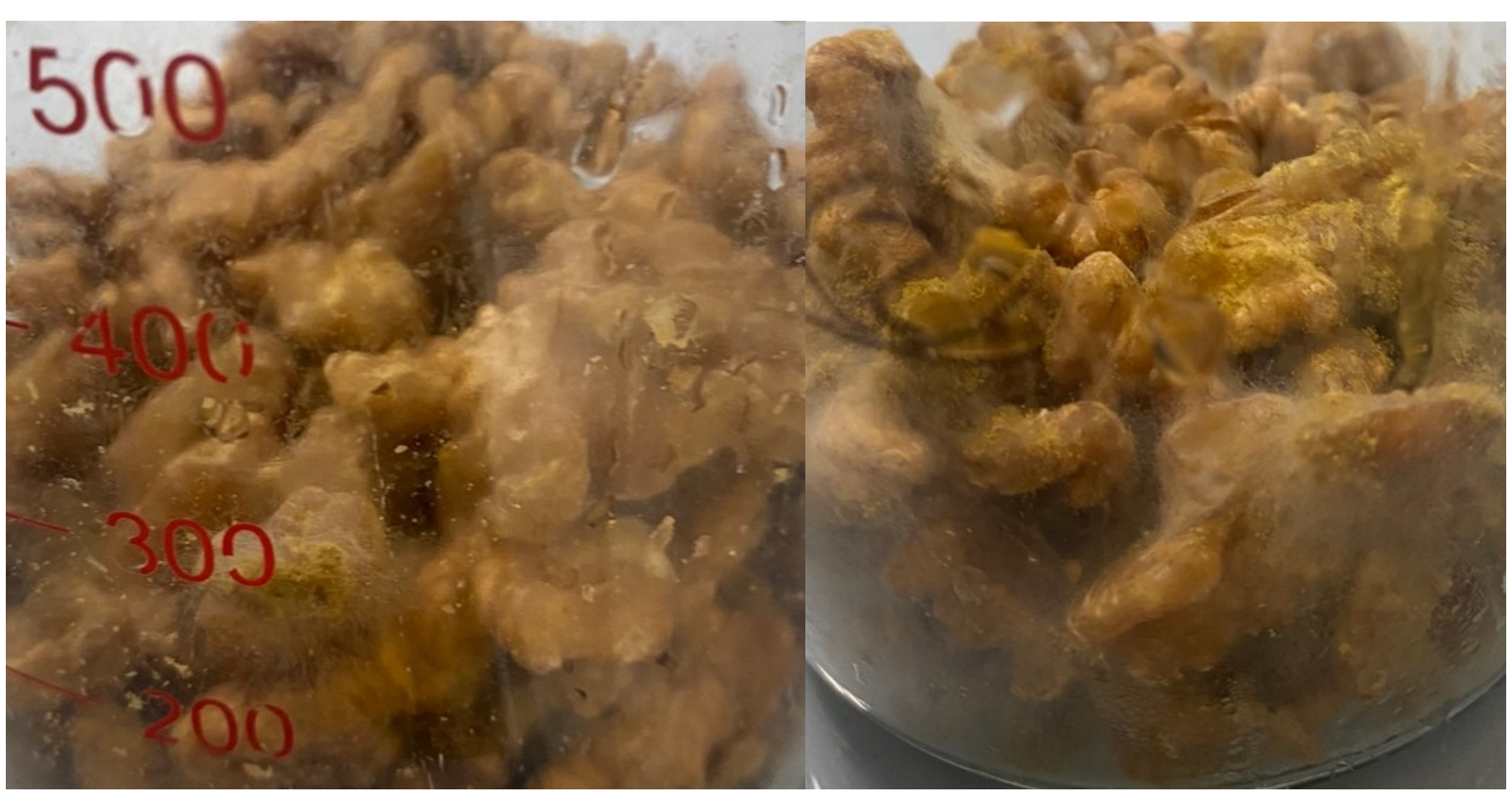

Supplementary figure 3-4 Walnut AF70 1 (left), walnut AF70 2 (right)

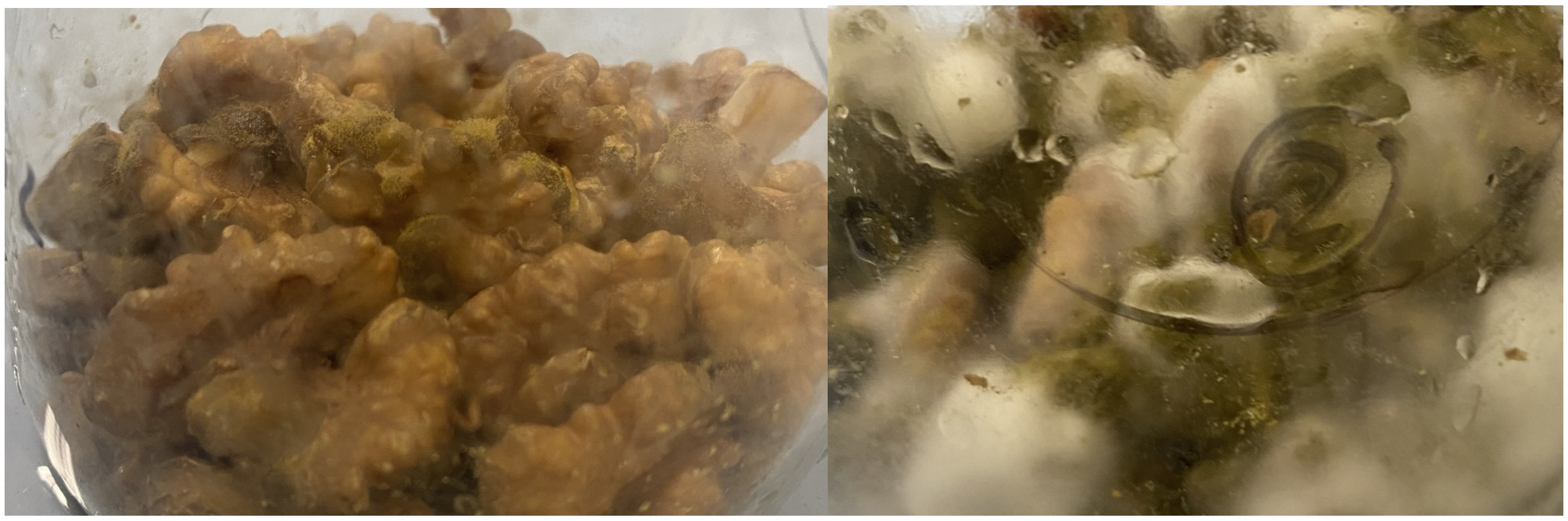

Supplementary figure 5-6 Walnut CBS 119.62 1 (left), walnut CBS 119.62 2 (right)

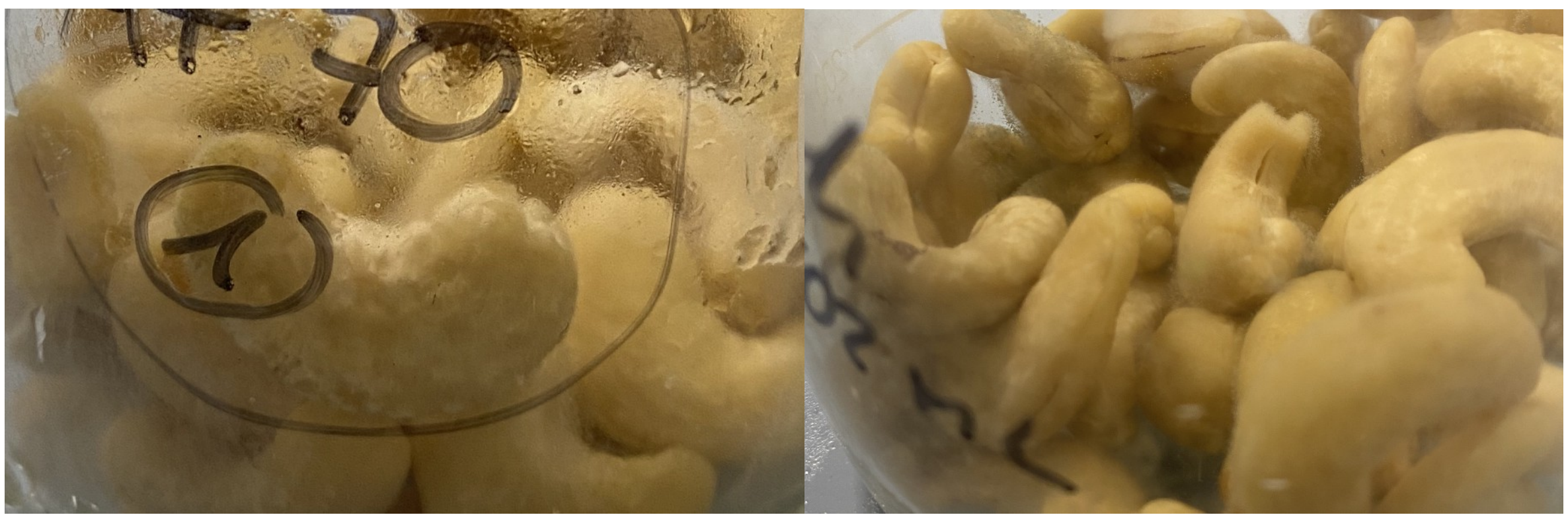

Supplementary figure 7-8 Cashew AF70 1 (left), cashew AF70 2 (right)

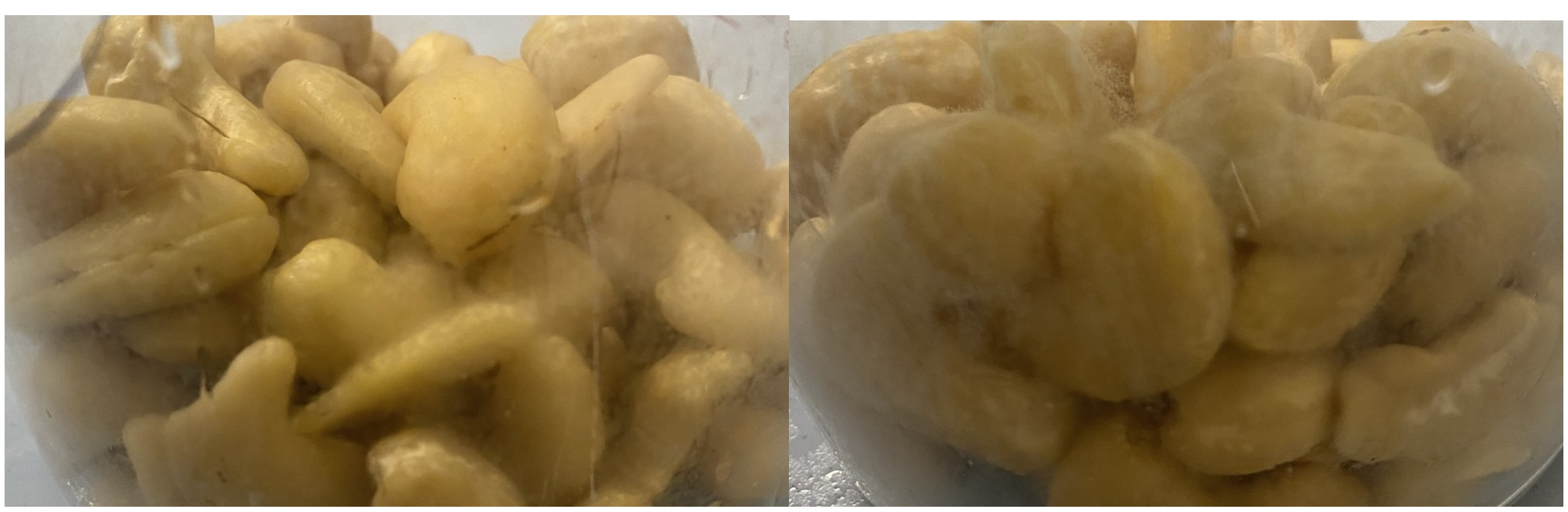

Supplementary figure 9-10 Cashew CBS 119.62 1 (left), cashew CBS 119.62 2 (right)

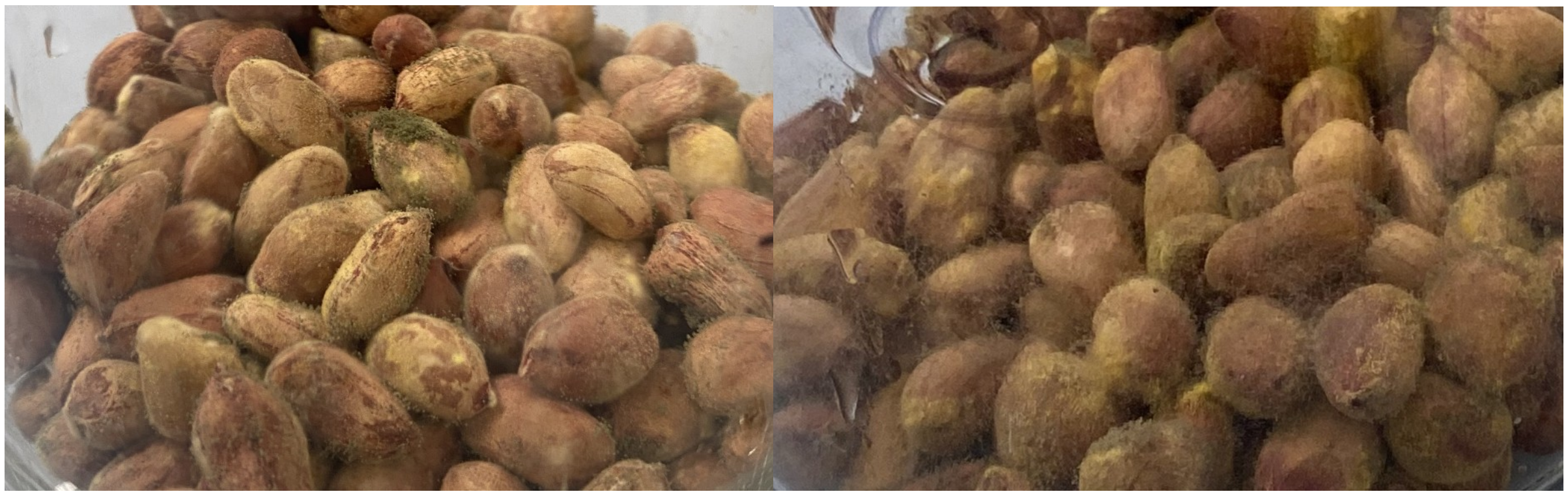

Supplementary figure 11-12 Peanut AF70 1 (left), peanut AF70 2 (right)

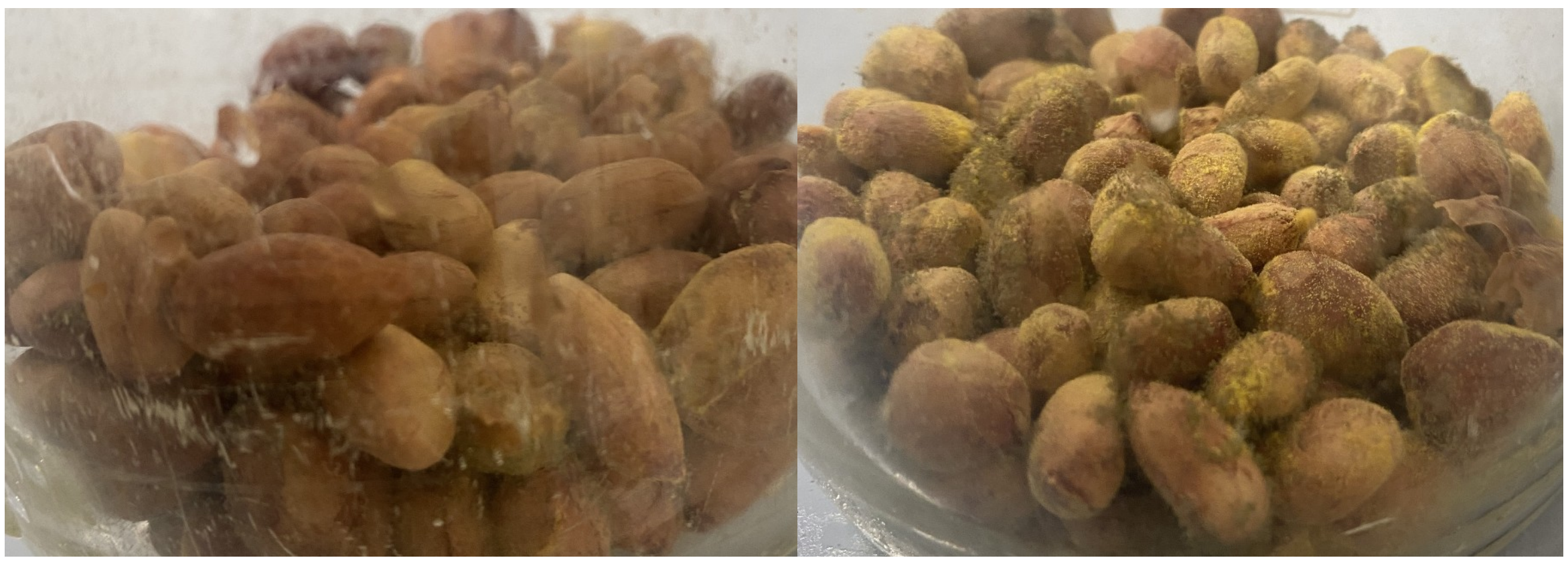

Supplementary figure 13-14 Peanut CBS 119.62 1 (left), peanut CBS 119.62 2 (right)

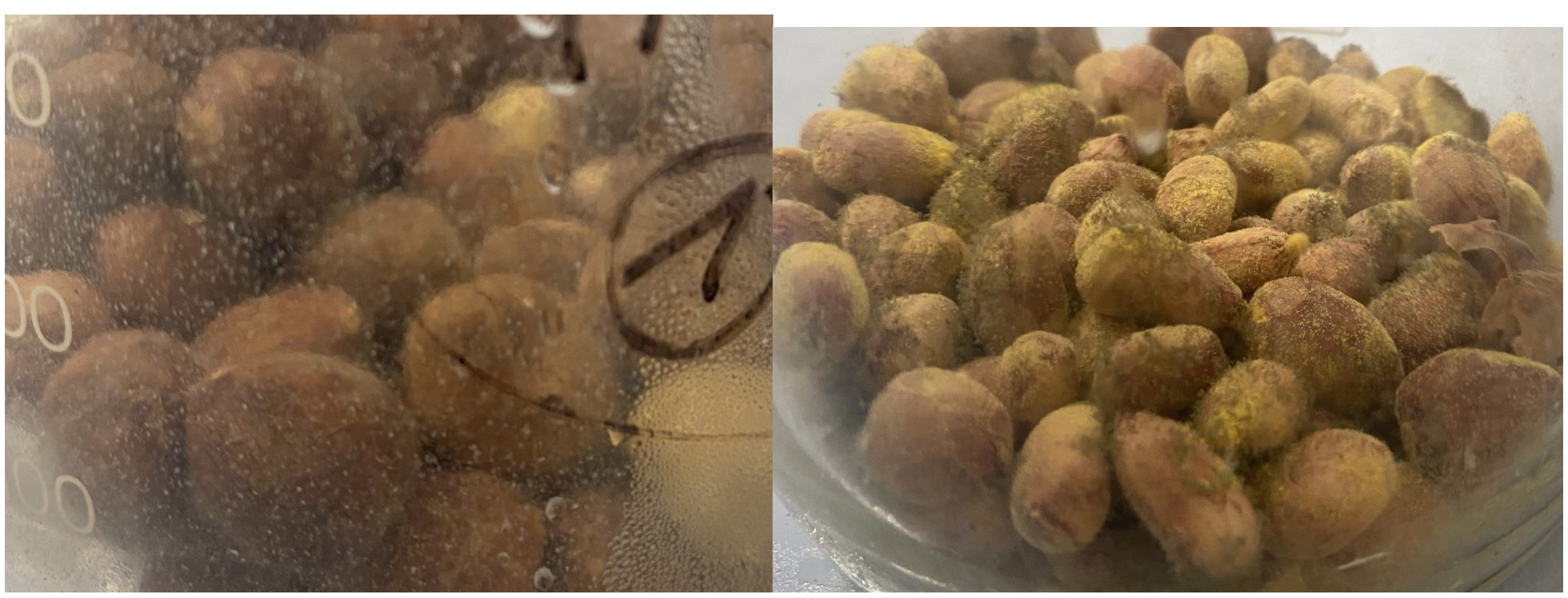

Supplementary figure 15-16 Hazelnut AF70 1 (left), hazelnut AF70 2 (right)

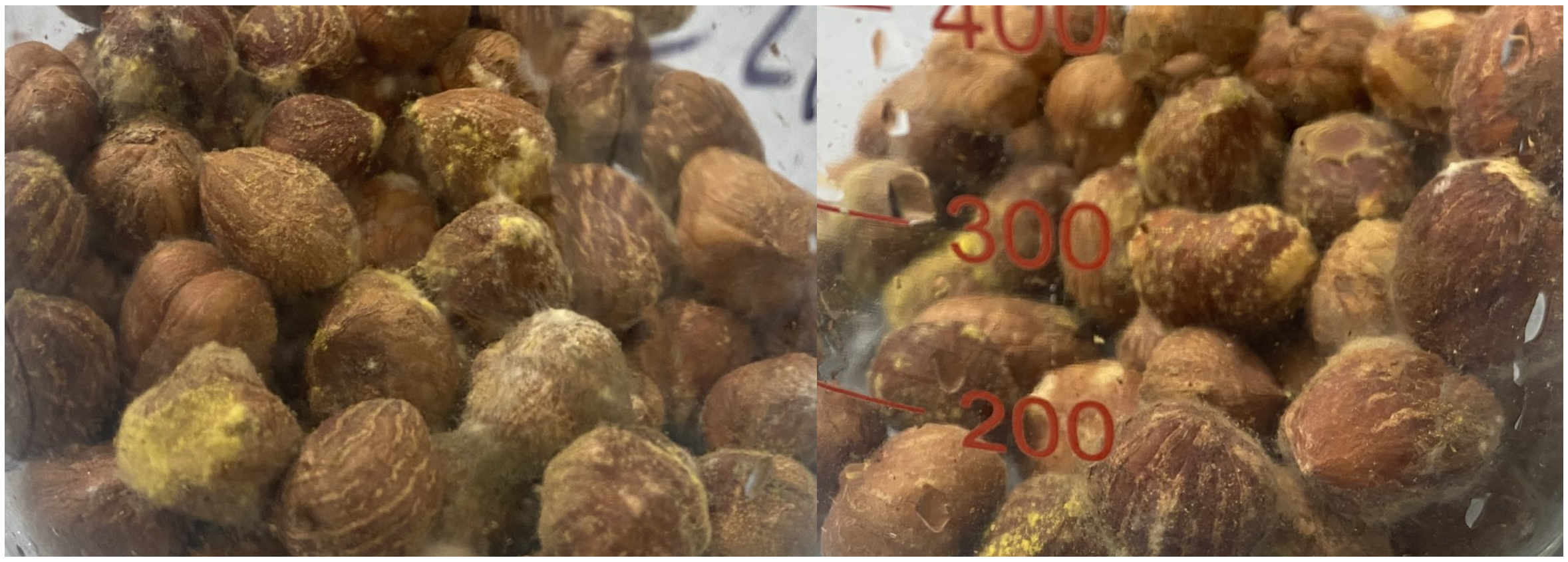

Supplementary figure 17-18 Hazelnut CBS 119.62 1 (left), hazelnut CBS 119.62 2 (right)

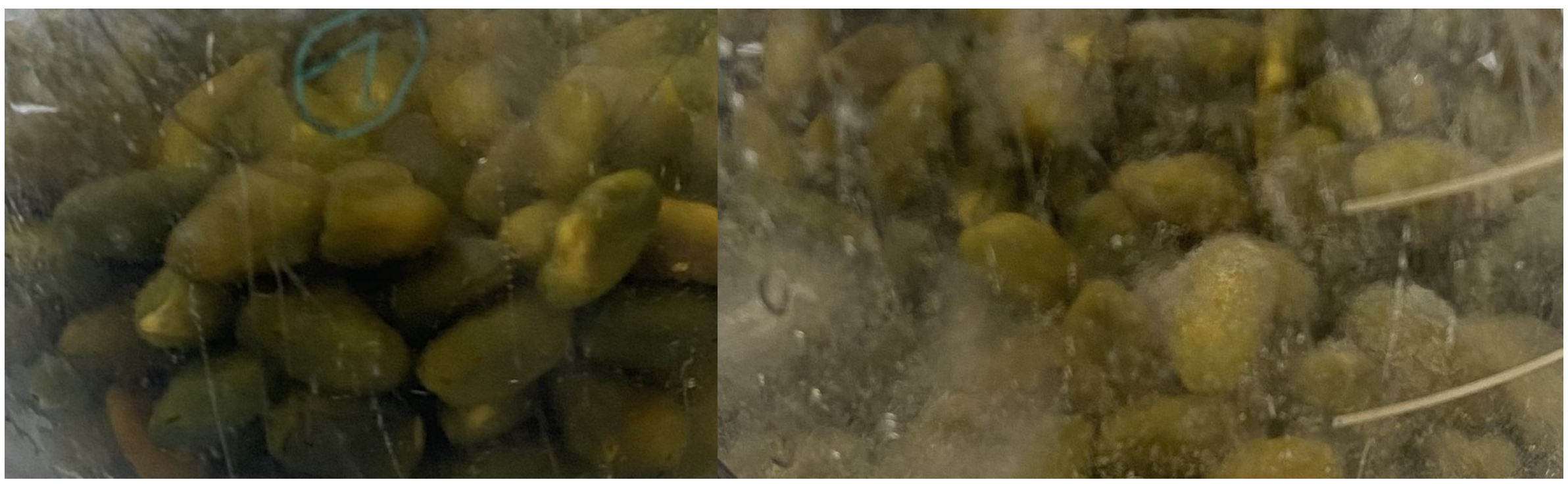

Supplementary figure 19-20 Pistachio AF70 1 (left), pistachio AF70 2 (right)

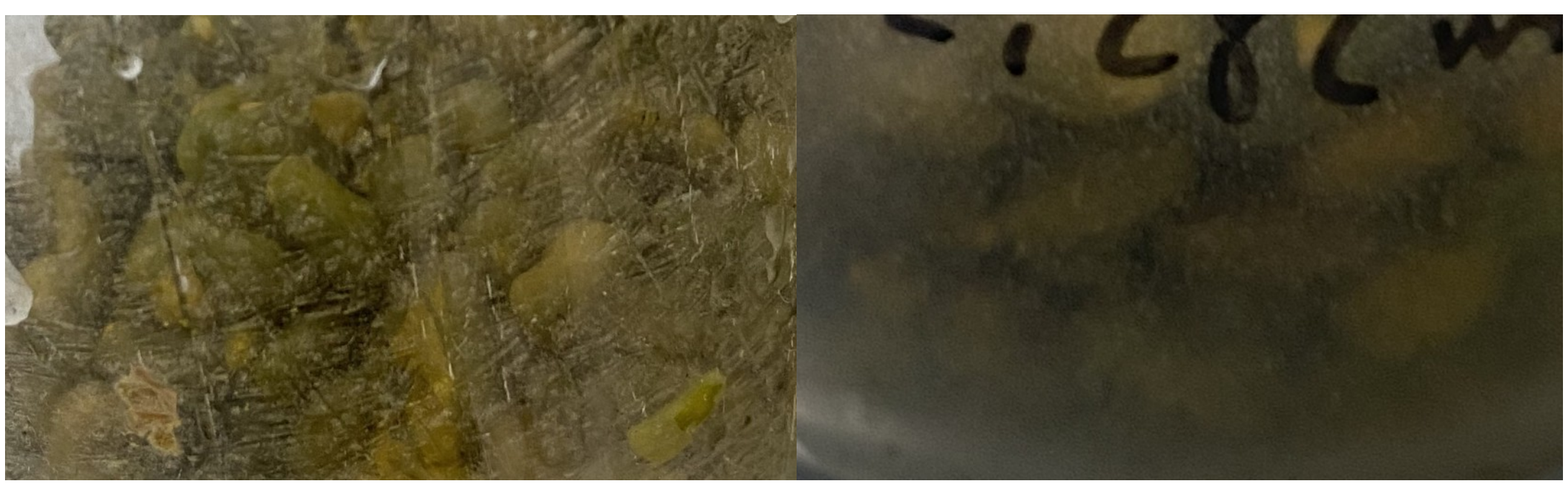

Supplementary figure 21-22 Pistachio CBS 119.62 1 (left), pistachio CBS 119.62 2 (right)
